# Formation and Preservation of Microbial Palisade Fabric in Silica Deposits from El Tatio, Chile

**DOI:** 10.1089/ast.2019.2025

**Published:** 2020-03-25

**Authors:** Jian Gong, Kimberly D. Myers, Carolina Munoz-Saez, Martin Homann, Joti Rouillard, Richard Wirth, Anja Schreiber, Mark A. van Zuilen

**Affiliations:** ^1^ Equipe Géomicrobiologie, Université de Paris, Institut de physique du globe de Paris, CNRS, Paris, France.; ^2^ Departamento de Geologia, FCFM, Centro de Excelencia en Geotermia de los Andes (CEGA), Universidad de Chile, Santiago, Chile.; ^3^ CNRS-UMR6538 Laboratoire Géosciences Océan, European Institute for Marine Studies, Technopôle Brest-Iroise, Plouzané, France.; ^4^ GeoForschungsZentrum, Section 3.5 Interface Geochemistry, D-14473, Potsdam, Germany.

**Keywords:** Silica sinter, Biosignature, El Tatio, Sheathed cyanobacteria, Experimental diagenesis, Mars

## Abstract

Palisade fabric is a ubiquitous texture of silica sinter found in low temperature (<40°C) regimes of hot spring environments, and it is formed when populations of filamentous microorganisms act as templates for silica polymerization. Although it is known that postdepositional processes such as biological degradation and dewatering can strongly affect preservation of these fabrics, the impact of extreme aridity has so far not been studied in detail. Here, we report a detailed analysis of recently silicified palisade fabrics from a geyser in El Tatio, Chile, tracing the progressive degradation of microorganisms within the silica matrix. This is complemented by heating experiments of natural sinter samples to assess the role of diagenesis. Sheathed cyanobacteria, identified as *Leptolyngbya* sp., were found to be incorporated into silica sinter by irregular cycles of wetting, evaporation, and mineral precipitation. Transmission electron microscopy analyses revealed that nanometer-sized silica particles are filling the pore space within individual cyanobacterial sheaths, giving rise to their structural rigidity to sustain a palisade fabric framework. Diagenesis experiments further show that the sheaths of the filaments are preferentially preserved relative to the trichomes, and that the amount of water present within the sinter is an important factor for overall preservation during burial. This study confirms that palisade fabrics are efficiently generated in a highly evaporative geothermal field, and that these biosignatures can be most effectively preserved under dry diagenetic conditions.

## 1. Introduction

Testing whether life ever emerged beyond the Earth depends on our ability to positively identify morphological features and chemical attributes that are uniquely indicative of life's fundamental processes (Cady *et al.*, 2003). In upcoming Mars missions and other future exploration, targets for the detection of life will be logically selected based on preservable properties of life in environments we know on the Earth (Neveu *et al.*, 2018), and, consequently, they require support of contextual and multi-scale observations in comparable environments that can be studied in detail.

The recent discovery of surface hydrothermal silica deposits on Mars (Squyres *et al.*, 2008; Ruff *et al.*, 2011) has renewed interest in hot spring-associated biosignatures on our own planet (Ruff and Farmer, 2016). Hot spring environments not only have the potential to preserve some of the earliest signatures of life 3.5 billion years ago (Djokic *et al.*, 2017), but they could also have contributed to the origin of life itself (Damer *et al.*, 2015).

Silica-rich hot spring systems contain microbial ecosystems that are complex, diverse, and well adapted to a wide range of temperatures along fluid discharge zones and channels (Jones *et al.*, 2003; Power *et al.*, 2018). Although microorganisms in these environments are not known to actively induce silica precipitation (Konhauser *et al.*, 2004), their presence is believed to provide a favorable template for the precipitation of silica (Cady and Farmer, 1996; Konhauser *et al.*, 2004; Benning *et al.*, 2005).

Silicification ultimately results in a multitude of geometric infills, replacements, casts, or molds around microbial cells that can be preserved and recognized as macroscopic, textural fabrics in sinter deposits (Preston *et al.*, 2008; Campbell *et al.*, 2015). Silicified fabrics, often described as *streamers*, *palisades*, *shrubs*, or *bushes* at millimeter-to-centimeter scales, have the potential to record detailed information regarding microbial communities and their environmental interactions (Walter, 1976; Walter *et al.*, 1996). The macroscopic size of these textural signatures also offers geologists and robotic instruments valuable first clues in accessing an unknown environment. Among these microbially induced silica structures, palisade fabrics are the most widely encountered in both modern and ancient hot spring deposits (Walter *et al.*, 1998; Campbell *et al.*, 2015; Djokic *et al.*, 2017).

Palisade fabric is characterized by closely packed, vertically stacked, micro-pillar structures that are usually bound by upper and lower lamina that are up to 5-mm thick (Lynne, 2012). Palisade fabrics are often found in low-temperature environments (<40°C) in modern hot springs and are associated with low relief sinter aprons dominated by sheet flows (Campbell *et al.*, 2015), as opposed to splash zones or channel flow areas where physical fluid forcing is stronger. Another distinctive character of palisade fabric is its primary porosity: Extensive pore structures are often regularly spaced and elongated, oriented primarily surface-normal along the principal fabric direction, giving rise to a filamentous morphology (Lynne, 2012: Fig. 4). Palisade fabrics are generally associated with the growth of sheathed, filamentous cyanobacteria (Walter, 1976; Cady and Farmer, 1996; Lynne and Campbell, 2003; Campbell *et al.*, 2015). Due to repeated alternation in filament directions, the same fabric has also been termed “stratiform stromatolite” (Jones *et al.*, 1998: Fig. 11; Konhauser *et al.*, 2004; Mata *et al.*, 2012).

The preservation potential of palisade fabric has been studied in detail, ranging from extant silicifying cyanobacteria in modern hot springs to silica sinters in opal-A, opal-CT, and quartz horizons in extinct hot springs of various ages (Lynne and Campbell, 2003; Campbell *et al.*, 2015). As was pointed out in the work of Campbell *et al.* (2015), preservation strongly depends on the particular conditions of early silicification and subsequent diagenesis. A particularly important condition, which has not received much scientific attention, is preservation of palisade texture in silica sinters under dry conditions such as in strongly evaporative environmental settings that could have prevailed on early Earth or possibly on early Mars.

The El Tatio Geothermal Field is located in the hyper-arid Atacama Desert region in the Northern Antofagasta Province (Region II), Chile, where high elevation (4300 m), low temperature, and low atmospheric pressure, combined with an evaporative and ultra violet (UV)-rich environment, make it one of the most valuable analog systems for the UV-inundated Archean and early Proterozoic Earth (Phoenix *et al.*, 2006). More recently, it has received considerable attention as an analogue for key target localities in upcoming Mars missions (Ruff and Farmer, 2016; Hays *et al.*, 2017).

**FIG. 1.**
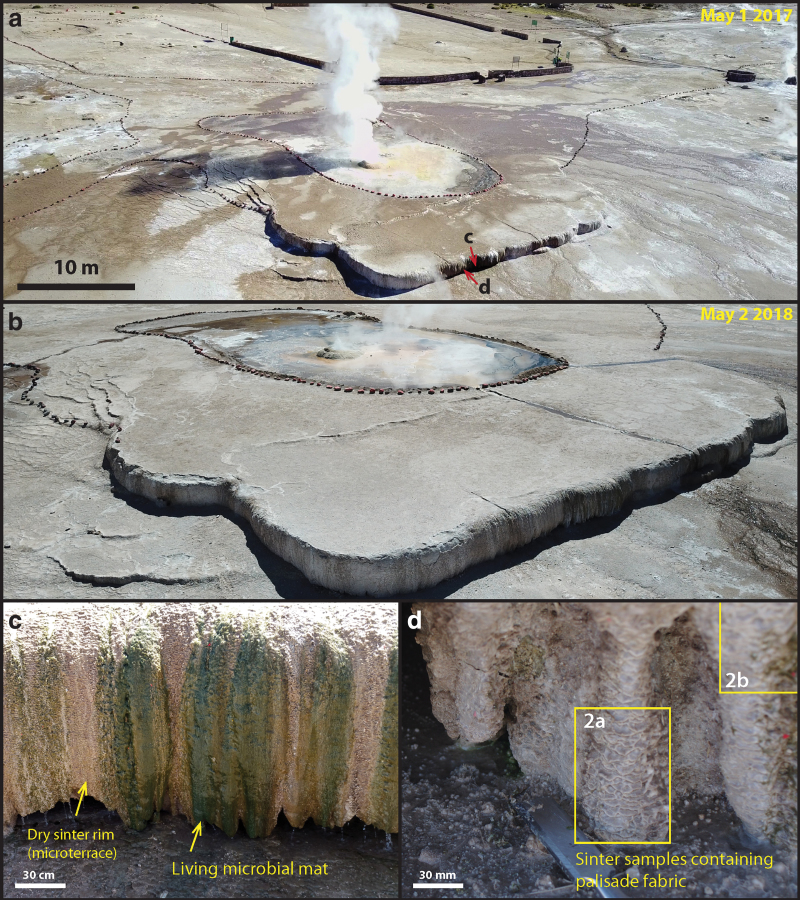
Regional overview of the Terrace Geyser, El Tatio Geothermal Field and sampling locations **(a)** Aerial image of the geyser, apron, and terrace taken by a drone on May 1, 2017. The arrow indicates the sampling location shown in **(c)** and **(d**). **(b)** Aerial image taken on May 2, 2018, demonstrating significant variations in wet**–**dry conditions of the sinter that are not seasonally controlled. **(c)** Green microbial mats found growing on the vertical surface of the sinter terrace where water is actively flowing. In nearby dryer areas, the sinter surfaces display a micro-terracette structure. **(d)** A detailed image of the micro-terracettes that are not covered by microbial mats. Boxed area indicating the samples collection site (corresponding to samples imaged in Fig. 2a and b).

The Terrace Geyser (local Spanish name: Géiser, GPS location: 22°19′51.7′′ S, 68°00′39.6′′W) is one of the largest geyser features at El Tatio Geothermal Field (Glennon and Pfaff, 2003: Upper Geyser Basin, geyser ID T25), where the active geyser currently discharges steam and fluids at the local boiling point of 86°C from a central vent, rapidly cools, forming a distinct, whitish splash zone with a diameter between 12 and 15 m (Fig. 1a). Sets of terrace structures form in the down-slope direction away from the splash zone, together creating a distinct sinter apron. At the rim of the sinter apron, densely populated microbial mats preferentially grow at the vertical walls of the fluid drop-off zone, where the water temperature cools below 30°C (Fig. 1c).

**FIG. 2.**
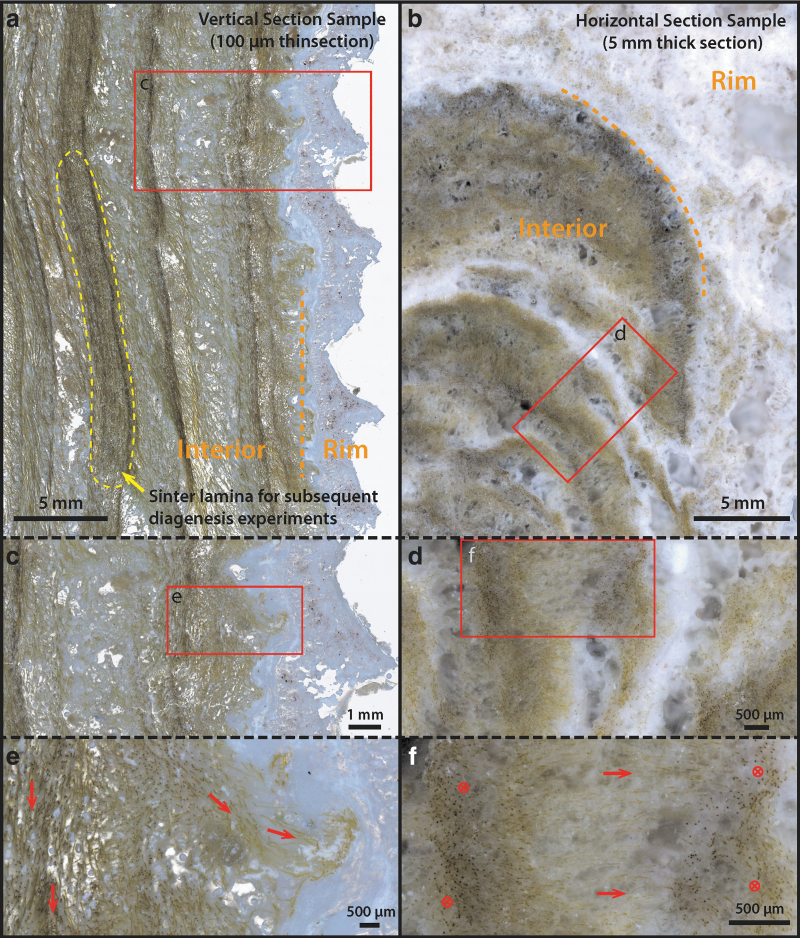
Cross-sectional microphotographs of the palisade fabric sample in vertical and horizontal cut directions. **(a)** Thin section (100 μm, vertical cut) overview, showing the whitish outer rim and laminated palisade fabric in the interior. The yellow dashed region indicates sinter lamina subsampled for diagenesis experiments. **(b)** Thick section (∼5 mm, horizontal cut) overview, showing the outer rim and the interior palisade lamina. Note the stromatolitic shape of the lamina in this cut direction and the whitish inter layers. **(c)** Zoom-in region from **(a)**. **(d)** Zoom-in region from **b**, showing significant porosity in the white and less-green layers **(e)** Further zoom-in region from **c**, showing alteration of filament alignment direction, from vertical on the left to horizontal on the right of the image (arrows). **(f)** A further zoom-in region from **d**, showing alteration of filament alignment direction, from in/out of the image plane to left/right in the middle of the image. Note the visibly higher porosity in the middle of the image. Alterations in filament alignment direction as well as the porosity generally characterize the palisade fabric lamina (arrows).

After photographs and descriptions of the Terrace Geyser from Jones and Renault (1997: Fig. 2; ET-4), Glennon and Pfaff, 2003 (geyser T25), and Phoenix *et al.* (2006: Fig. 1b, c), significant past variations in the geyser's activity may be assessed: In 2003, it was described to perpetually spout 2–3 m with 5-m-high eruptions and was covered by broad sheets of water; whereas now it spouts only 0.5 m with 1–1.5-m eruptions, and many parts of the terrace are dormant (Fig. 1a, b). These observations bring novel perspectives in studying the formation mechanism of palisade fabric with respect to wet**–**dry cycles of the sinter, as well as understanding the preservation pathways of biosignatures unique to the highly evaporative environment.

Here, we trace the path of degradation of cyanobacteria into the silica sinter and show how trichomes are lost while sheaths form the scaffolding for emerging silica fabrics. Combined DNA sequencing and stable isotopic analysis (carbon and nitrogen) were applied to these sinter samples to better understand the degradation of microbial mats at various stages after silicification, once they were no longer undergoing active carbon assimilation by photosynthesis. To further understand the effect of diagenesis on the preservation potential of sheathed organisms in palisade fabrics, beyond what can be observed under field conditions, we artificially accelerated the degradation process through laboratory experiments at elevated temperature and pressures. The entire process of degradation of cyanobacteria-based palisade fabric was carefully described by using confocal laser scanning microscopy (CLSM), scanning electron microscopy, and transmission electron microscopy (SEM and TEM respectively).

## 2. Materials and Methods

### 2.1. Sampling area and sampling protocols

Samples of sinter and surface microbial mats were collected from the Terrace Geyser during a field campaign in late April 2017. At the edge of the large terrace structure (Fig. 1a), where the surface is wet, dark green microbial mats were actively growing from the sinter surface, many forming columnar structures that expand outward and overlap each other (Fig. 1c). Due to the extreme day**–**night air temperature contrast caused by the desert climate, the temperature of the fluid reaching the terrace rim changed throughout the day as well. At the sampling location (GPS: 22°19′52.1′′ S, 68°00′39.3′′ W), the fluid temperature was measured as 15°C at dawn and 30°C at noon with a thermometer (Fisher NC9065215, Accuracy: 1°C); whereas in the nearby dry sinter structures, where hot spring effluents were not actively flowing, surface temperature was measured as 5°C at dawn and 30°C at noon with a noncontact infrared thermometer (Nubee NUB8250, accuracy: ±2% or 2°C). Here, the columnar structures did not show green mats at the exterior, but they displayed a rough, micro-terrace surface texture (Fig. 1c, d).

Hard sinter samples were obtained with a chisel that was sterilized on-site with 70% ethanol. Samples were placed immediately in sterile bags (VWR Ref# BURK5344), transported, and stored at 0–4°C (unfrozen) in thermal insulating bags until reaching the lab. Within 1 week of sampling, a subset of these samples was separated and frozen at −20°C to preserve DNA and isotopic signals. To avoid textural changes from freezing, all diagenesis experiment materials originated from the unfrozen portion of the same sample, shown in Figs. 1d and 2a. Free-living surface microbial mat samples (Fig. 1c, 1–2 m away from the hard sinter samples shown in Fig. 1d) intended for microscopic analysis were collected with a sterile tweezer, fixed on site with 2% glutaraldehyde, stored and transported at 0–4°C, and analyzed within a week's time.

### 2.2. DNA sequencing and bioinformatics

Sinter samples were sub-divided into the outer-most crusted rim (ET17-L14_Rim) and laminated interior layers underlying the crust (ET-L14_Interior) (Fig. 2a). DNA was extracted and sequenced in duplicate (ET17-L14_Rim) and triplicate (ET17-L14_Interior), depending on the amount of raw material available. Genomic DNA was extracted with a Qiagen™ DNeasy^®^ PowerLyzer^®^ PowerSoil^®^ kit (Qiagen Sciences, Germantown, MD) following the manufacturer's protocols, with two modifications to the initial recovery step to enhance DNA recovery from the low-biomass samples: (1) PowerBead (0.1 mm) tubes were replaced with PowerBead Glass (0.5 mm) tubes (Qiagen) to increase the physical destruction of palisades sinter material and increase cell exposure to the reagents, and (2) 0.3 mg/L proteinase K (Qiagen) was added to bead tubes containing sample material and sodium dodecyl sulfate to remove proteins and improve the quality of recovered DNA. Bead tubes were incubated at 55°C for 30 min before and after vortexing, to increase proteinase K activity and aid protein unfolding for digestion.

Community diversity and taxonomic identification of prokaryotic microorganisms were assessed by using short- and full-length 16S rRNA amplicon surveys on Illumina MiSeq (Illumina, Inc., San Diego, CA) and Pacbio sequel (Pacific Biosciences, Menlo Park, CA) platforms, respectively. Sequencing and preliminary taxonomy-dependent bioinformatics were performed at MRDNA (Molecular Research Laboratory LLC Shallowater, TX) by using bacterial tag-encoded FLX amplicon (bTEFAP^®^) sequencing technology (Dowd *et al.*, 2008). Short reads (Illumina) were amplified from the V4–5 region of the 16S rRNA gene (515f-926r) (Walters *et al.*, 2016) to an average depth of 100k reads/sample. Following standard quality control removal steps (Caporaso *et al.*, 2010), an average of 70k nonchimeric sequences per sample were aligned to cultured organisms and database-accessioned environmental sequences by using the most recent Greengenes alignment database v. 13.8 (DeSantis *et al.*, 2006). Although the samples were found to contain a fraction of rare and unknown organisms, only high-confidence matches to known sequences are discussed here.

### 2.3. Optical microscopy

Free-living surface microbial mat samples were imaged directly with an Olympus BX-41 biological microscope. Hard sinter samples were prepared for both thin (100 μm) and thick (∼5 mm) sections. These samples were first air-dried in a sterile laminar fume hood for 3–7 days while monitoring weight loss. After drying, a subset of samples was mounted in epoxy resin (EpoxiCure 2; Buehler) under three to five vacuum cycles to remove air bubbles.

These samples were further cut and polished with a diamond blade lubricated with pure mineral oil (CAS: 8042-47-5, Sigma-Aldrich: 415080010) to prevent dissolution of salts. Selected sections were scanned with a Zeiss Axio Zoom.V16 motorized stereo microscope (IBPS, UPMC) by using combined transmitted and reflected light to obtain large area images at 1000 × magnification (corresponding to ∼1 μm/pixel). Higher resolution optical images in selected areas were also obtained with the Olympus BX-41 microscope.

### 2.4. CLSM analysis

Free-living surface microbial mat samples (prefixed with 2% glutaraldehyde on-site) and air-dried, epoxy-embedded thin and thick sections of sinter samples (without fixation) were analyzed by CLSM. CLSM was performed at IBPS, UPMC with a Leica TCS-SP5 inverted confocal microscope (Leica Microsystems Heidelberg GmbH, Manheim, Germany) by using a 63 × (NA 1.4, oil) Plan-Apochromatic objective.

The Leica TCS-SP5 employs a spectrophotometric detection system (the lambda/wavelength scan function) that can perform fluorescent emission scans over the entire visible spectrum (400–800-nm) by using a motorized slit placed in front of the photomultiplier. Emission scans were performed by using the 488-nm laser line as excitation source during each scan, which has been characterized *in vivo* to primarily excite phycobilin and carotenoid pigments in cyanobacteria (Vermaas *et al.*, 2008).

Each image sequence was obtained by scanning the same *x* − *y* optical section using a bandwidth of 10 nm and at 5-nm steps (scan range for the 488 nm laser excitation was set from 498 to 750 nm). This technique is commonly employed to study auto-fluorescent properties of unknown natural photosynthetic pigments, allowing a complete characterization of the excitation–emission response of the photosynthetic apparatus (Roldán *et al.*, 2004; Supplementary Data S1). During CLSM image acquisition, 2D images were obtained throughout the sample depth, forming a 3D stack. Differential interference contrast (DIC) optics was also employed during 3D stacking so that the optical view is available as a monochromatic image. These 3D-stacked images were then projected back to 2D by using the maximum intensity projection algorithm available in the software (Leica Application Suite X version 3.3, Leica Microsystems). Selected CLSM 3D stacks (without the DIC component) were deconvoluted by using the software suite Huygens Professional (Scientific Volume Imaging B.V.), which improved the lateral spatial resolution exceeding the Rayleigh limit (Cotte *et al.*, 2010).

### 2.5. SEM preparation and analysis

Free-living microbial filaments fixed with 2% glutaraldehyde were gently washed in water, placed directly on double-sided carbon tape on an SEM stub, air-dried, and sputter-coated with gold before imaging. Hard sinter samples (not embedded in epoxy resin) were first air-dried, then artificially fractured into 3–5-mm chunks with a sterile knife or plier, and subsequently placed on carbon tape and gold-coated. SEM was performed at the IPGP PARI analytical platform with a Zeiss GeminiSEM 500, FEG-SEM at 3–6 keV and a working distance of 5 mm. Energy-Dispersive X-ray spectroscopy (EDX) spectra and maps were obtained separately with a Zeiss EVO MA-10, at 15 kV and 12-mm working distance.

### 2.6. Focused ion beam milling and TEM analysis

Thin foils (15 × 10 × 0.1 μm) for TEM analysis were prepared at GeoForschungsZentrum Potsdam (GFZ; Germany) by focused ion beam (FIB) milling using an FEI FIB 200 equipped with a Ga-ion source. FIB-sputtering was performed on a Pt-coated area on a silica sinter thin section; subsequently, thin slides were lifted out, placed on a standard Cu TEM grid, and covered with lacey carbon. Details of this FIB preparation technique are given in Wirth (2009). TEM analysis was carried out at GFZ, Germany, with an FEI TecnaiG2 F20 X-TWIN equipped with a Gatan Imaging Filter GIF (Gatan Tridiem, Gaatan, CA), an EDAX X-ray analyzer, and a Fishione high-angle annular dark-field (HAADF) detector. The analyses were made by using a field emission gun emitter set at an acceleration voltage of 200 kV.

### 2.7. Artificial diagenesis experiments

Two sets of artificial diagenesis experiments conducted in this study were designed to accelerate diagenesis in the temperature regime 120–250°C. A final high-temperature step at 300°C was performed to study strong alteration at the onset of metamorphism (Winter, 2009). Although these experiments employ elevated temperatures and pressures as tools to inform the possible effects of diagenesis on the sinter deposit, they do not necessarily mimic geological conditions or time. Therefore, these experiments should be considered in an approach to study the relative degradation of different components of microbial cells and their surrounding silica matrix, and not as simulations of true geological alteration.

In the first set of experiments, untreated samples were subjected to the same *p* = 250 bar pressure but at varying temperatures *T* = 165°C, 250°C, and 300°C for 72 h; whereas in the second set of experiments, untreated or air-dried samples were subjected to progressively higher temperature and pressure conditions in four stages, with the first two stages lasting 5 days (120 h) and the last two stages lasting 3 days (72 h): (1) *T* = 120°C and *P* = 1 kbar, 5 days, (2) *T* = 165°C and *P* = 1.5 kbar, 5 days, (3) *T* = 250°C and *P* = 2.5 kbar, 3 days, and (4) *T* = 300°C and *P* = 3 kbar, 3 days. At each step, samples were retrieved and analyzed. The conditions for the first set of experiments were designed to repeat previous artificial fossilization and diagenesis experiments (Oehler and Schopf, 1971; Alleon *et al.*, 2016) that had some success in preserving cellular structures, whereas the second set of experiments is novel, as it follows a realistic burial scenario in which samples are subjected to T/P conditions of the modern geothermal gradient of the Earth at 25°C/km-depth-of-burial (Supplementary Data S2).

For all experiments, millimeter-sized chunks of the palisade sinter were broken off with a sterile tweezer or plier and placed directly in pure gold tubes (2.5 mm OD and 2.3 mm ID, 10–15 mm long) (Wieland Edelmetalle, Germany). Dry samples for the second batch of experiments were prepared by air-drying in a sterile laminar flow hood for ∼2 weeks while monitoring weight loss, as air-drying caused weight reduction of up to a third of the original sample weight due to water loss. After sample filling, all gold capsules were flushed with a stream of pure Ar gas for 30 s, after which the open tube ends were immediately crimped and weld-sealed with an electric fine point welding system (PUK U4, Lampert Werktechnik GmbH). Duplicate or triplicate samples were produced and analyzed to ensure reproducibility.

The sealed gold capsules were subsequently loaded in batches into custom-designed autoclaves (Top Industrie, Vaux-le-Pénil, France). Pure Ar gas was used as the pressure medium. During experiments, autoclaves were first put under pressure with pure Ar gas before heating was applied, to minimize pressure-induced heating during pumping in closed volumes. In the first set of single-step experiments, the samples were directly brought to target temperature and pressure conditions within less than 30 min, whereas the cooling step after each completed experiment took ∼5 h to reach room temperature. Similarly, for the second set of progressive alteration experiments, each increase in temperature was performed within 30 min, and cooling after completion of the last step was accomplished within ∼5 h.

Before and after each experiment, each gold capsule was individually weighed and compared to ensure no leakage of the capsule had occurred during the experiment. After each experiment, gold capsules were cut open directly with mechanical tools and the inner materials were carefully placed directly on double-sided carbon tape on SEM stubs. After sputter coating with gold, the samples were analyzed by SEM. For TEM analysis, these materials were first embedded in epoxy resin (EpoxiCure 2, Buehler), polished, and coated with carbon before FIB milling was performed.

### 2.8. Carbon and nitrogen isotopic analysis

Organic carbon and nitrogen isotopic analysis were performed at the Pôle Spectométrie Océan (PSO, Brest, France). Two palisade sinter samples corresponding exactly to the DNA sequence analysis (Section 2.2) (Fig. 2a: rim and interior), plus a third palisade sinter sample corresponding to the interior portion (Fig. 2b) were analyzed. These samples were previously frozen at −20°C to preserve isotopic signals, and they were processed at the same time with the DNA samples. After thawing, the samples were immediately air-dried in a sterile laminar flow for 2 days, then crushed into a fine powder with an agate mill grinder, decarbonated overnight with 6 M HCl, and finally warmed at 80°C for 2 h in a fume hood. Residues were rinsed with Milli-Q water and centrifuged several times until they approached a neutral pH. 25–30 mg of decarbonated sample material was loaded into a tin capsule and analyzed with a Thermo Scientific Delta V Plus mass spectrometer coupled to a Flash 2000 elemental analyzer.

## 3. Results

### 3.1. Morphological descriptions of sheathed microbial filaments in sinter samples

Sinter samples at the edge of terraces, when viewed in cross-section, are composed of brown-to-green-colored, dense filamentous microorganisms embedded in a porous silicified matrix (Fig. 2a, b). Two sinter samples were analyzed in detail: One was sectioned in the vertical direction (Figs. 1d and 2a), whereas the other was sectioned along the horizontal direction (Fig. 2b).

Microscopically, the vertically sectioned sample revealed filamentous structures forming in alignment and at an angle to lamination, constituting a characteristic palisade fabric (Fig. 2a, c); whereas the horizontally sectioned sample displayed layers of contorted, stromatolitic lamina (Fig. 2b). In both samples, the sinter structures were divided into two distinct regions: a whitish outer rim forming micro-terracettes at the exterior (Figs. 1d and 2a) and an interior region consisting of the same filamentous microorganisms that compose the laminated mats (Fig. 2a).

Samples for artificial diagenesis experiments (Section 2.7) were subsampled in the interior region, in a single layer highlighted by the yellow dashed area in Fig. 2a, where microbial filaments appeared to be particularly dense. At the scale of microbial populations (Fig. 2c–f), except at the outer crusted rim, the interior lighter-green laminae are visibly more porous (Fig. 2b–f) in comparison with the denser green layers. Within the dense green lamina (Fig. 2e, f: arrows), microbial filaments are generally found parallel to lamination, aligning with the downward local flow direction. This is in contrast with the whitish and more porous laminae, within which microbial filaments are predominantly aligned perpendicular to lamination (Fig. 2e, f: arrows). Here, the orientation of filaments appears to correlate with elongated pores that run perpendicular to lamination (*e.g.*, Fig. 2d).

### 3.2. Free-living sheathed cyanobacterial mats at the exterior of the sinter

**FIG. 3.**
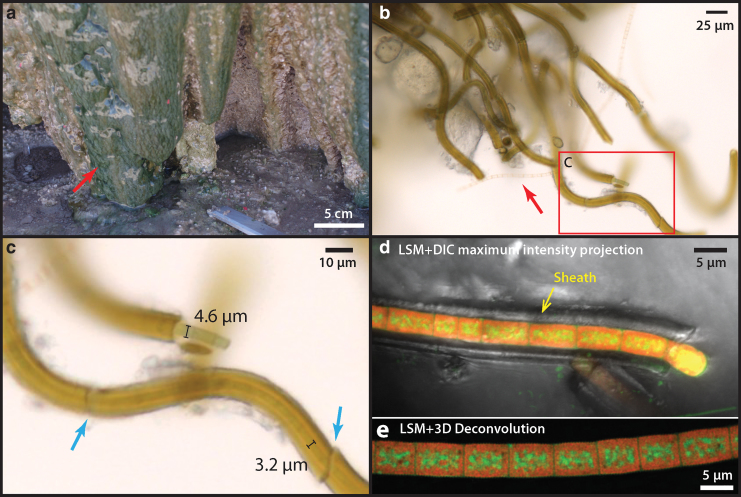
Free-living sheathed cyanobacteria from the wet region of nearby closely associated sinter surface. **(a)** Microbial mats showing a reticulate morphology under the sheet flow. Note the vertically aligned reticulate ridges (dense green lines). **(b)** Optical micrograph of a microbial layer from mats in **(a)** showing sheathed filamentous (boxed area detailed in **(c)**) cells as well as an unsheathed, semi-transparent filament (arrow). **(c)** Details of the filaments in **(b)** showing variable diameter of cells within the sheath. Note the breakage (arrows) of the fragile sheath when handled under the microscope. **(d)** CLSM image of a filament excited with 488-nm laser, overlaid with an optical image taken with the DIC optics. Note that the sheath did not fluoresce. **(e)** CLSM stacked image processed with 3D deconvolution, showing localization of pigments. Green and Red are false colors corresponding to the 530 and 660 nm peaks in the fluorescence spectra (Fig. 4). Green color is interpreted as carotenoids (β-carotene), and red color is interpreted as thylakoid-membrane-bound phycobilins. CLSM, confocal laser scanning microscopy; DIC, differential interference contrast.

Free-living microbial mats at the exterior of the sinter show a reticulate texture growing under a thin sheet of flowing water (Fig. 3a: arrow). The local ridges of these reticulates (denser green lines) show some degrees of alignment with the downward fluid flow direction (Fig. 3a), indicating that local mat growth is influenced by fluid forcing. These mat surfaces, however, are generally smoother than the nearby dried sinter crusts that display a microterrace morphology (*e.g.*, Fig. 1d). Microorganisms collected from these exterior surfaces possess a sheathed filamentous morphology, although in places some unsheathed cells can also be found with empty interiors (Fig. 3b: arrow). Microscopic observations indicate that the diameters of external sheaths are between 6 and 10 μm, whereas the filamentous cells within sheath are typically between 3 and 5 μm (Fig. 3b, c), consistent with filamentous cyanobacteria such as *Leptolyngbya* sp., which are also known to thrive in the current temperature regime of the terraces (5–20°C) in the presence of moderate to high salinity (Komárek, 2007).

Single cyanobacterial trichomes that occur within their own sheaths are uniserate (unbranching). Cells are isodiametric (1.5–3 times greater length than diameter), and trichomes are arranged in chains of filaments (Fig. 3c). The actively dividing ends of the trichomes, which extend beyond the sheath, are conical and rounded, and no heterocysts or akinetes (resting cells) were observed (Fig. 3c).

**FIG. 4.**
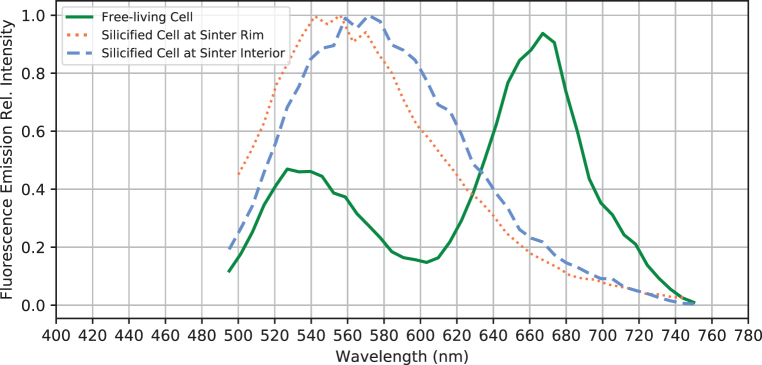
Fluorescence emission spectra of selected free-living and fossilized sheathed cyanobacteria. Note the loss of the 670-nm peak after the free-living cyanobacteria are entombed within the sinter.

CLSM showed that cells auto-fluoresced when excited by the 488-nm laser, producing two pronounced emission peaks centered at 530 and 660 nm (Fig. 4). The pigments responsible for these fluorescence signals are interpreted as β-carotene (Fig. 3d, e: green, corresponding with the 530 nm peak in Fig. 4) and phycobilins (Fig. 3d, e: red, corresponding with the 660 nm peak in Fig. 4). These fluorescence signals are consistent with the fluorescence wavelengths and location of the common cyanobacterial accessory pigments known as carotenoids (β-carotene) and phycobilins (Schluchter and Glazer, 1999). The deconvoluted CLSM image shows that phycobilins are preferentially localized at the thylakoid membrane structure of the cell (shown as red in Fig. 3e), whereas carotenoids are more concentrated toward the cell interior (green color in Fig. 3e).

**FIG. 5.**
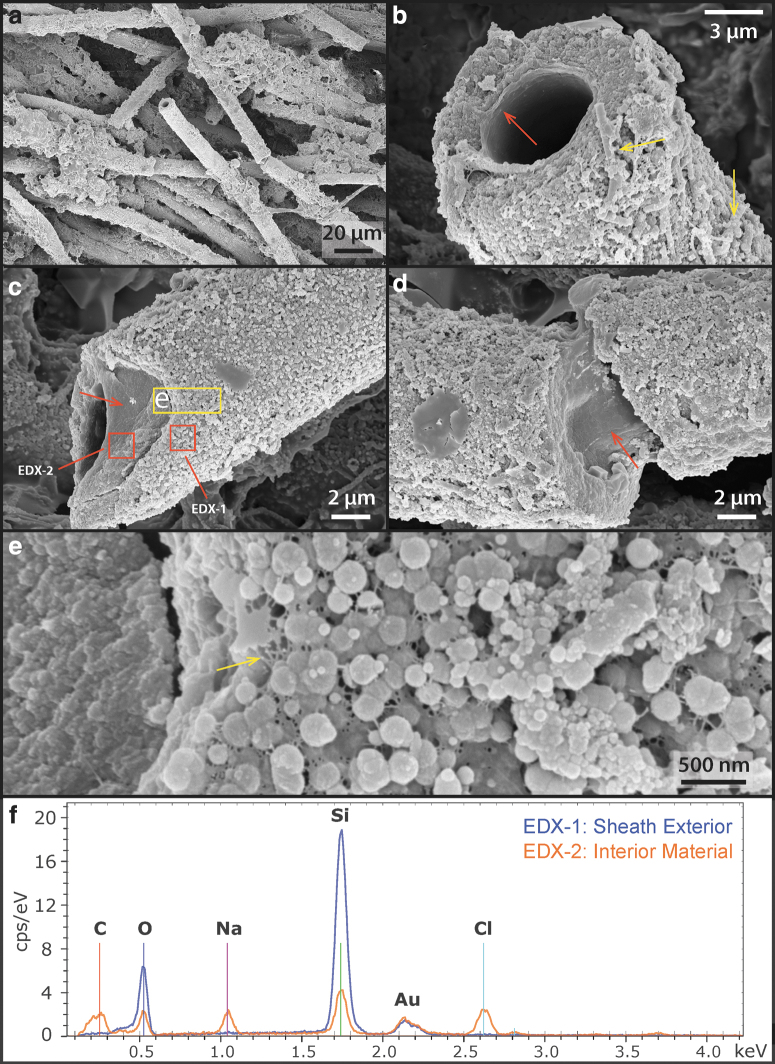
SEM images of the free-living sheathed cyanobacteria. **(a)** Overview image of sheathed filaments. **(b)** Close-up view of a sheath structure, showing a smooth internal surface texture (red arrow) and some small filaments or rods at the exterior of the sheath (yellow arrows). **(c)** Close-up view of another sheathed structure. The internal smooth-textured material has an EDX spectrum (EDX-2 in **f**), indicating that it consists of cellular organic carbon. The outer surface of the sheath consists of predominantly amorphous silica (EDX-1 in **f**), indicating that early silicification has already occurred. **(d)** Another view of a sheathed filament, also showing internal organic material. **(e)** High-resolution image of the sheath surface (from **c**), showing spherical silica structures coating nanometer-sized filamentous structures (arrow). **(f)** EDX spectra corresponding to studied regions in **(c)**. EDX; SEM, scanning electron microscopy.

Analysis by SEM shows that the interior surfaces of the sheaths (Fig. 4b–d: red arrows) are universally coated with a smooth, nonsiliceous organic layer (Fig. 5f: EDX-2 showing the carbon peak); whereas the sheath exteriors consist of a fine, porous network of filamentous structures (Fig. 5e: yellow arrow) that are covered by amorphous silica spheres ranging in size from a few nm to ∼100 nm. These tiny filamentous structures are morphologically similar to the fine fibrillar meshwork of dehydrated extracellular polymeric substances (EPS) described in other studies of hot spring microbial communities (Handley *et al.*, 2008; Handley and Campbell, 2011).

Frequently, smaller diameter (∼500 nm) and shorter filaments were also found at the exterior of sheaths (Fig. 5b: yellow arrows). These could represent hormogonia of the parent *Leptolyngbya* sp., which were produced in response to environmental stress (*e.g.*, episodes of rapid mineral precipitation under evaporative conditions). These observations together suggest that although the organisms were still alive, early silicification had already occurred, beginning at the exterior of the cyanobacterial sheath, creating a silica cast.

### 3.3. Early silica-encrusted cells at the sinter rim

Detailed microscopic and EDX chemical analysis indicate that the outer rim region of the palisade sinter sample (Figs. 1d and 2a) comprises a 3–4-mm-thick crust of mixtures of opal-A silica, halite, and other granular material: Halite is concentrated at the tips of the micro-terracettes and also at the exterior surface of the sinter crust (Supplementary Fig. S1). Individual grains have variable elemental composition, and they are generally enriched in K, Ca, or Al, but not Cl (Supplementary Fig. S1), suggesting that they represent wind-blown sand from surrounding andesitic and rhyolitic ignimbrite deposits of the greater El Tatio region (Healy and Hochstein, 1973; Tassi *et al.*, 2005). The outer rim region shows significant porosity, with cavities increasing in size from 30 μm in the outer halite-concentrated zone, up to 100–500 μm size toward the deeper interior (Supplementary Fig. S1).

**FIG. 6.**
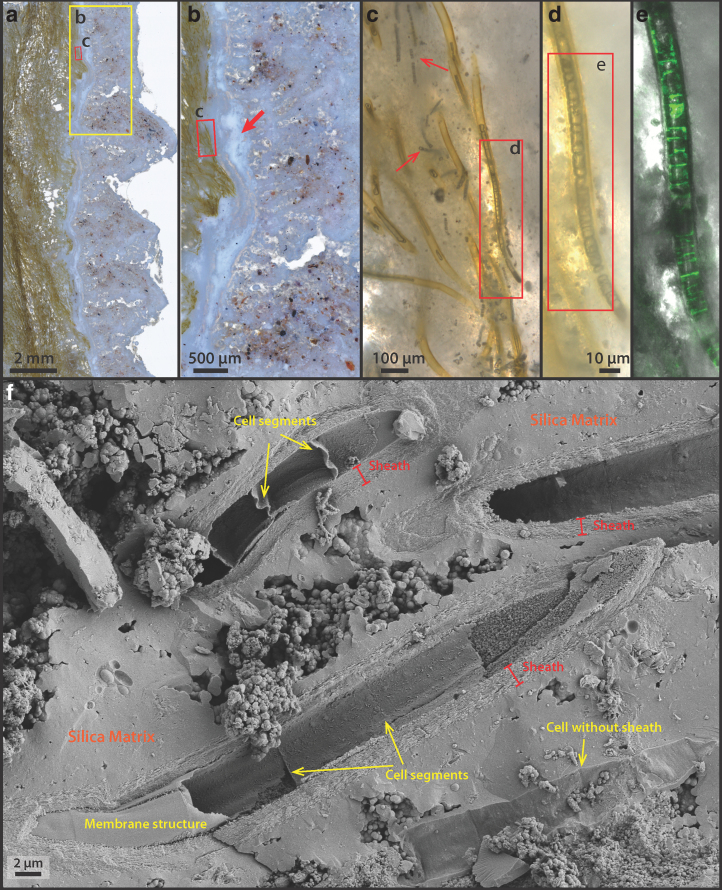
Detailed image of the palisade sinter rim and silicified, sheathed microbial filaments immediately below the rim toward the interior of the sinter. **(a)** Overview image of the sinter rim. **(b)** Close-up image of the interface between the rim and green microbial filaments. Note the milky white layer at the base of the rim immediately overlaying the microbial filaments (arrow). Sand grains are also found throughout the outer rim structure, whereas no signs of microbial cells can be identified. **(c)** Zoom-in image of the sheathed microbial filament. Most sheathed filaments appear hollow, but occasionally some internal structures are found to be preserved **(d)**. Some short-segmented, unsheathed filamentous cells are present in close proximity to the sheathed filaments (arrows). **(d)** Close-up view of the sheathed filament, which contains some internal segmented structures. **(e)** CLSM of the filament from **(d)** overlain with optical DIC image, showing that these segmented structures respond to the 488-nm laser excitation. The corresponding emission spectrum was reported in Fig. 4. **(f)** SEM image of the microbial community in an unembedded sample (corresponding to **c**), showing segmented filamentous cells within the sheath, detailed sheath morphology, and an unsheathed filamentous cell casted by silica (arrows). The sheath structure was measured to be consistently between 1 and 2 μm in thickness.

Microorganisms do not occur in the outer most white crusts (Fig. 6a), but they are clearly present at the base of the outer rim region underneath a solid whitish horizon (Fig. 6b: arrow). These observations indicate that the outer rim region of the palisade sinter sample experienced dry and evaporative conditions that allowed halite to precipitate. It appears that the outer rim represents an intensely dry period of the sinter growth, where silica and salts were rapidly deposited. Under these circumstances, the sheathed cyanobacterial mat community was rapidly entombed before this evaporative sinter growth process took over. Specific microbial communities adapted to dry conditions may have been present in this evaporative rim, but any trace of them has since been destroyed by strong UV radiation and oxidation.

Directly underneath the outer rim crust (Fig. 6a, b), sheathed and nonsheathed microbial filaments are present in close proximity (Fig. 6c: arrows), with the sheath structure often appearing hollow, occasionally shown with trichomes preserved (Fig. 6a–d). Internal cellular materials have been lost, possibly due to physical destruction during sinter drying, paired with decomposition by associated, sheath-dwelling heterotrophic organisms. The remains of these previous cellular membrane structures, however, have a fluorescence signal in response to the 488-nm laser excitation (Fig. 6e).

In direct comparison to the free-living cyanobacteria described earlier (Section 3.2), these membrane structures only possess a single fluorescence emission peak centered around 540–560 nm (Fig. 4), indicating that the previously interpreted thylakoid-bound phycobilins pigments had been degraded and de-localized from previously intact thylakoid membranes (observed in Fig. 3d, e). In addition, SEM images show preserved cell membrane segments within the silicified sheaths, demonstrating that unsheathed as well as sheathed cells can leave a cast within the silica matrix (Fig. 6f).

### 3.4. Completely silicified cells in the interior of the sinter sample

**FIG. 7.**
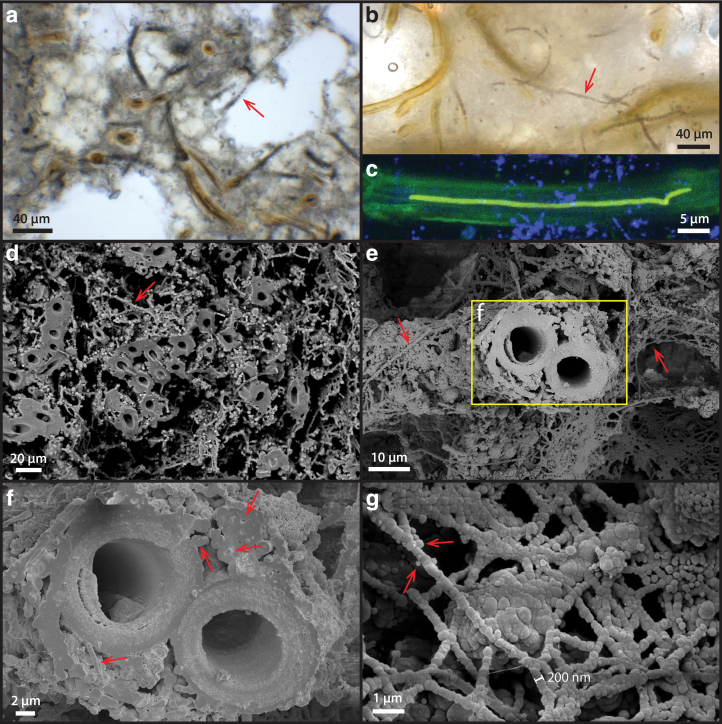
Detailed image of the palisade sinter interior (corresponding to Fig. 2a: dashed region). **(a)** Sheathed as well as unsheathed filamentous cells at the sinter interior. **(b)** Similar, sheathed and unsheathed cells. **(c)** Shrunken filamentous cells within the sheath that also respond to the 488-nm laser excitation. The blue colors are reflections of 405-nm laser from a separate channel, aiming at illuminating the background matrix material. The corresponding fluorescence emission spectrum of the sample is reported in Fig. 4. **(d)** SEM image of the sinter interior, showing a sheathed framework interspaced with other smaller filamentous structures. **(e)** An alternative view of the interior, showing a silicified sheath covered by smaller filamentous structures (arrows). **(f)** Zoom-in image from (**e)**, showing detailed interior structure of these smaller filaments: Some of these smaller filaments have an interior structure, or are hollow, whereas others are completely filled (arrows). **(g)** Exterior view of these smaller filaments (<500 nm), showing that they form a network structure while they are covered by spherical silica structures (arrows).

The interior of the palisade sinter sample (Fig. 2a: yellow dashed region) consists of bundles of silicified sheaths forming a framework structure and a porous silica matrix surrounding the framework (Fig. 7a, d, and e). Abundant unsheathed and partially degraded filamentous cells can be found in the matrix (Fig. 7a, b: arrows). Sheaths are hollow or contain shrunken remnants of filamentous cells, with diameters between 1 and 2 μm (Fig. 7b, c) and cell shapes reminiscent of those in the living mats (Figs. 3 and 6). These shrunken cells within sheaths also fluoresce in response to the 488-nm laser excitation, displaying an emission spectrum forming a single broad peak centered at 560–570 nm wavelength (Fig. 4).

Some unshrunken, unsheathed filamentous cells, 3–4 μm in diameter were also present in the interior (Supplementary Fig. S3). These cells also fluoresce with the 488-nm laser, indicating that they could be remains of cyanobacteria similar to the unsheathed filamentous cells at the sinter rim (Supplementary Fig. S2).

SEM images also revealed that in addition to sheathed filaments, much thinner filamentous structures ranging from 200 nm to about 1 μm are present, filling the space in between sheaths (Fig. 7d), as well as coating the exterior surface of existing silicified sheaths (Fig. 7e). These enigmatic small filaments often do not have a well-defined internal structure: Some contain a solid spherical center, whereas others contain small holes or are featureless (Fig. 7f: arrows).

These filaments also branch out in connected networks while being coated by silica spheres of various sizes (Fig. 7g: arrows). These small, silica-encrusted filaments could represent degraded or dried out EPS, similar to the EPS filaments observed in palisade fabrics in silicified cyanobacterial mats of hot springs at Orakei Korako (Campbell *et al.*, 2015). Alternatively, some of the larger diameter filaments (close to 1 μm) could represent the shrunken counterparts of the unsheathed cyanobacterial trichomes that are seen in the outer rim. The absence of a CLSM fluorescence signal (Supplementary Fig. S3b, e, and f), however, rules out this possibility. Therefore, it is also possible that these small filaments represent other groups of small unsheathed microorganisms that are present in the interior of the sinter.

**FIG. 8.**
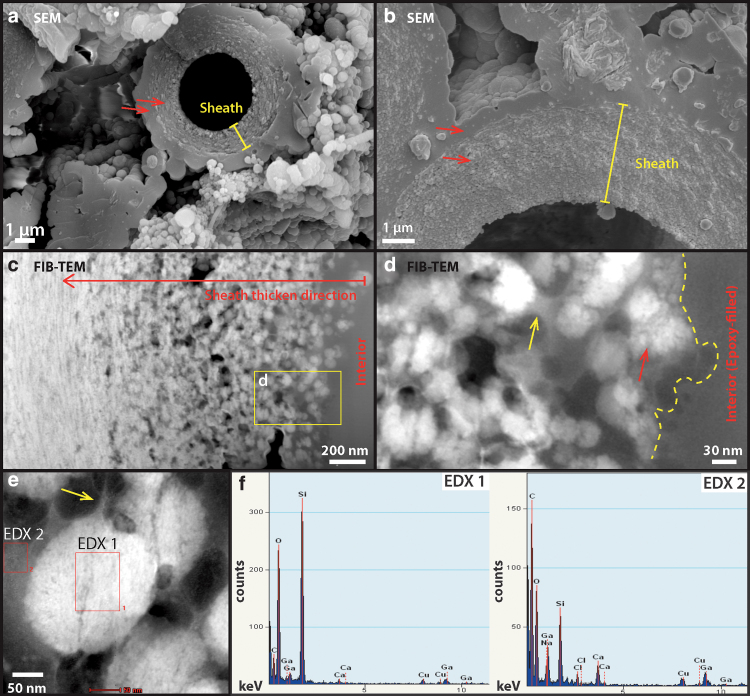
High-resolution SEM and TEM analysis of silicified sheath structures at the interior of the sinter. **(a)** One- to two-micrometer-thick, rough-textured sheath structure is overlain by a smooth-textured silica (arrows) at the exterior. **(b)** Close-up view of the transition from rough-textured sheath to smooth-textured silica (arrows). **(c)** TEM image of FIB-prepared foil (100 nm thick) reveals a clear change of silica morphology from ∼50 nm spheres at the interior to sheets-like toward the exterior. **(d)** Zoom-in image of the silica spheres, showing a clotted texture (red arrow) as well as an amorphous material that connects and surrounds these spheres (yellow arrow). **(e)** EDX chemical analyses of the silica spheres (EDX-1 in **f**), confirming that they consist of only silica, and the surrounding matrix material (EDX-2 in **f**), which was interpreted as dehydrated extracellular organics.

The silicified sheath structures themselves show granular-textured interiors coated by a layer of smooth silica at the exterior (Fig. 8a, b: arrows). TEM images of an FIB-milled foil that is cross-cutting a sheath show that the interior consists of ∼20–50-nm-sized silica spheres that fill a porous network of EPS (Fig. 8c, d). These silica particles gradually change in morphology from spherical to platy/sheet-like filling toward the outer rim of the sheath, whereas the overall structural porosity is significantly reduced (Fig. 8c, d).

The interior silica spheres display a clotted internal texture (Fig. 8d: red arrow) whereas the spheres themselves are coated by an amorphous material (Fig. 8d: yellow arrow), shown as darkened areas in the resulting HAADF TEM image. EDX spectra indicate that this material is enriched in carbon, sodium, calcium, and chlorine in contrast to the silica spheres (Fig. 8f), suggesting the co-occurrence of organic material and salt.

Due to incomplete penetration of the epoxy resin (Fig. 8c, d) and the fact that both the lacey carbon TEM support film and the epoxy resin are not known to contain notable amounts of sodium, calcium, or halite salts, the EDX signal presented in the amorphous films coating silica spheres is interpreted as dehydrated EPS. This observation suggests that the presence of EPS exerted a significant control on the morphogenesis of silica spheres, templating their formation from spherical (close to cyanobacterial cells) to lenticular or platy shapes (away from cells). Consequently, the thickness, as well as internal structural transitions of the sheath record the interplay between biological EPS production and silica precipitation from evaporating, silica- and salt-saturated fluids.

### 3.5. Microbial community structure shown by 16S rRNA gene sequencing

**FIG. 9.**
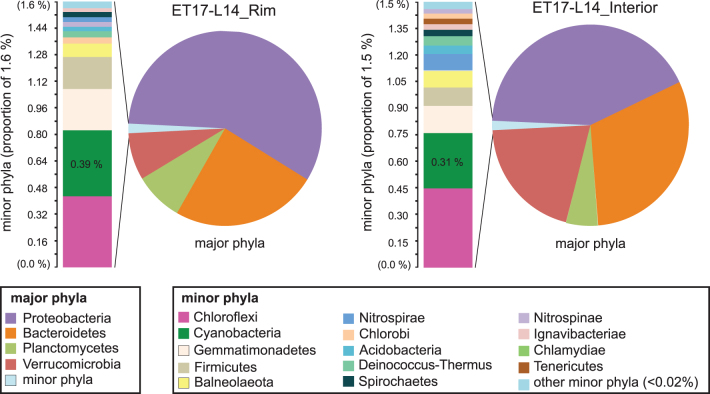
Phylum-level community membership and diversity in palisade samples ET17-L14_Rim and ET17-L14_Interior. Major phyla (pie charts) are dominated by four groups, totaling 98.4% of sequences (rim, upper left) and 98.5% (interior, upper right). Detailed phyla table is provided in Supplementary Data S3. The remaining 1.5–2.1% of sequences comprising the set of minor phyla in each sample is displayed in adjacent bar charts. Phyla are color-coded (key, bottom).

The microbial community structure of the outer rim crust and the relatively older interior of the sinter (Fig. 2a) were compared at the phylum and generic levels (Fig. 9; also see Supplementary Data S3). Composition of the rim and interior communities is largely similar, with a few notable distinctions. Although the represented major phyla are the same, differences in the relative abundances of major phyla are seen: Members of phylum Proteobacteria are less abundant in the older, interior sample, whereas taxa within the Bacteroidetes and Verrucomicrobia phyla are more abundant in the interior of ET17-L14 relative to the rim.

Similarly, slight differences are seen in the relative abundances of minor phyla (Fig. 9). Overall, minor phylum-level membership is also the same between these groups, with one distinction: The phylum Tenericutes (Fig. 9, lower left, brown bar) is present in the interior but not seen in the crust. Cyanobacteria, which dominate the outer microbial mats, made up a very small proportion of amplicon libraries generated on both Illumina and Pacbio platforms in both of the communities derived from lithified palisades material, and they were found to comprise less than 0.5% of total sequences in both the individual sub-samples and averaged sequences, pooled from two separately extracted and sequenced sub-samples of the rim, and three from the interior. Members of the Chloroflexi also comprised a small proportion of community membership with similarly low (0.35–0.45%) abundance.

At the generic taxonomic level, the outer rim was dominated by Proteobacteria *Algiphilus* sp. (Table 1: 24% of total taxonomically identified sequences); however, in the interior (Table 2), *Algiphilus* sp. had decreased to only 4%, whereas *Opitutus* sp. (Verrucomicrobia phylum) 11%, *Yeosuana* (Bacteroidetes phylum) 8%, and *Neorickettsia* (Proteobacteria phylum) 8% had arisen to make up the community.

**Table 1.** ET17-L14_Rim

Phylum	Class	Genera	%
Bacteroidetes	Flavobacteriia	*Gelidibacter*	8.74
Bacteroidetes	Flavobacteriia	*Yeosuana*	4.23
Bacteroidetes	Flavobacteriia	*Owenweeksia*	2.34
Bacteroidetes	Flavobacteriia	*Maribacter*	2.19
Bacteroidetes	Flavobacteriia	*Flavobacterium*	1.93
Bacteroidetes	Flavobacteriia	*Bizionia*	1.51
Planctomycetes	Planctomycetia	*Planctomyces*	4.97
Planctomycetes	Phycisphaerae	*Phycisphaera*	2.30
Proteobacteria	Alphaproteobacteria	*Paracoccus*	3.71
Proteobacteria	Alphaproteobacteria	*Rickettsia*	2.76
Proteobacteria	Alphaproteobacteria	*Rhodothalassium*	2.35
Proteobacteria	Alphaproteobacteria	*Parvibaculum*	1.79
Proteobacteria	Alphaproteobacteria	*Roseovarius*	1.19
Proteobacteria	Alphaproteobacteria	*Rubrimonas*	1.10
Proteobacteria	Alphaproteobacteria	*Porphyrobacter*	1.07
Proteobacteria	Gammaproteobacteria	***Algiphilus***	**24.02**
Proteobacteria	Gammaproteobacteria	*Marinobacter*	3.76
Proteobacteria	Gammaproteobacteria	*Methylobacter*	3.31
Proteobacteria	Gammaproteobacteria	*Silanimonas*	2.44
Verrucomicrobia	Verrucomicrobiae	*Verrucomicrobium*	6.23
Rare genera (<1% total abundance)			18.00

Dominant genera and their proportional abundance of total taxonomically identified sequences (%) present in the sample. The single most abundant genus in bold.

**Table 2.** ET17-L14_Interior

Phylum	Class	Genera	%
Bacteroidetes	Flavobacteriia	*Yeosuana*	8.12
Bacteroidetes	Flavobacteriia	*Flavobacterium*	5.14
Bacteroidetes	Flavobacteriia	*Psychroflexus*	4.23
Bacteroidetes	Flavobacteriia	*Salegentibacter*	2.13
Bacteroidetes	Flavobacteriia	*Owenweeksia*	2.17
Bacteroidetes	Flavobacteriia	*Maribacter*	1.83
Bacteroidetes	Flavobacteriia	*Bizionia*	1.75
Bacteroidetes	Flavobacteriia	*Algibacter*	1.42
Planctomycetes	Planctomycetia	*Planctomyces*	3.33
Planctomycetes	Planctomycetia	*Isosphaera*	1.27
Proteobacteria	Alphaproteobacteria	*Neorickettsia*	8.05
Proteobacteria	Alphaproteobacteria	*Rickettsia*	3.93
Proteobacteria	Alphaproteobacteria	*Paracoccus*	2.61
Proteobacteria	Alphaproteobacteria	*Rhodothalassium*	1.35
Proteobacteria	Alphaproteobacteria	*Parvibaculum*	1.12
Proteobacteria	Gammaproteobacteria	*Algiphilus*	4.27
Proteobacteria	Gammaproteobacteria	*Marinobacter*	6.71
Proteobacteria	Gammaproteobacteria	*Pseudoalteromonas*	1.40
Proteobacteria	Gammaproteobacteria	*Pseudomonas*	1.31
Proteobacteria	Gammaproteobacteria	*Alcanivorax*	1.05
Verrucomicrobia	Opitutae	***Opitutus***	11.02
Verrucomicrobia	Opitutae	*Coraliomargarita*	9.00
	*Rare genera (<1% total abundance)*		17.00

Dominant genera and their proportional abundance of total taxonomically identified sequences (%) present in the interior portion of the ET17-L14 sample. The single most abundant genus in bold.

Cyanobacteria only make up a small fraction of the community abundance at the rim as well as the interior of the palisade sinter sample (Table 3), even though cyanobacteria visibly dominate the biomass observed in the surface mats and images of the rim and interior. This suggests that these phototrophic communities begin degrading once they are dried; phototrophic metabolisms are greatly reduced after entombment in silica and salt; and the DNA sequence abundance in the community shifts to reflect more recently active organisms that degraded the original DNA and cell biomass, leaving behind the recalcitrant sheath and other silicified structures. However, among the small subset of sequences that were taxonomically identified as cyanobacteria, *Leptolyngbya* sp. comprise the majority of each sample, which is consistent with the strong morphological evidence for *Leptolyngbya*-like cell and filament size, division, growth, and morphology observed in the mats, rim, and interior of the sinter (Figs. 3, 5, and 6) (Castenholz *et al.*, 2001). No sequences matching *Calothrix* sp. or other Type IV cyanobacteria (*e.g.*, Rivulareaceae family) were identified.

**Table 3.** ET17-L14_Rim and ET17-L14_Interior

Type (sub-class)	Order	Genus	Crust		Interior	
			% tot	% cy	% tot	% cy
Cyanobacteria I	Chroococcales	***Synechocystis***	0.13	**32.21**	0.004	1.25
Cyanobacteria III	Oscillatoriales	***Leptolyngbya***	0.097	**24.50**	0.098	**31.25**
Cyanobacteria III	Oscillatoriales	***Plectonema***	0.061	15.44	0.086	**27.50**
Cyanobacteria V	Stigonematales	*Fischerella*	0.057	14.43	0.080	25.42
Cyanobacteria III	Oscillatoriales	*Arthrospira*	0.007	1.68	0.013	4.17
Cyanobacteria I	Chroococcales	*Gloeocapsa*	0.015	3.69	0.003	0.83
Cyanobacteria I	Chroococcales	*Cyanobacterium*	0.007	1.68	0.009	2.92
Cyanobacteria III	Oscillatoriales	*Schizothrix*	0.009	2.35	0.008	2.5
Cyanobacteria I	Chroococcales	*Synechococcus*	0.003	0.67	0.008	2.5
Cyanobacteria IV	Nostocales	*Calothrix*	0.004	1.01	0	0
Cyanobacteria I	Chroococcales	*Acaryochloris*	0.003	0.67	0.003	0.83
Cyanobacteria II	Pleurocapsales	*Hyella*	0.001	0.34	0.003	0.83
Cyanobacteria II	Pleurocapsales	*Prochlorococcus*	0.001	0.34	0	0
Cyanobacteria III	Oscillatoriales	*Phormidesmis*	0.001	0.34	0	0
Cyanobacteria III	Oscillatoriales	*Phormidium*	0.003	0.67	0	0

Comparison of the most abundant cyanobacterial genera, by proportional (%) abundance compared with total sequence counts (average of all available sequences and samples), and proportional abundances of each genus (%) compared with total counts of Phylum Cyanobacteria. The two most abundant genera in each sinter zone are shown in bold.

### 3.6. Carbon and nitrogen isotope analysis

At the outer rim of the palisade fabric sinter, the extracted organic carbon (Table 4) shows a δ^13^C of −23.18 ± 0.11‰, consistent with the presence of cyanobacteria (O'Leary, 1988; Zerkle *et al.*, 2005). Toward the interior of the sinter, however, two samples show less negative carbon isotope ratios (−13.77‰ ± 0.11‰ to −15.78‰ ± 0.11‰), a sharp increase of 8–10‰. The δ^15^N was −1.72 ± 0.15‰ at the rim, consistent with cyanobacteria that primarily fix N_2_ from the atmosphere (Wada *et al.*, 2012; Berrendero *et al.*, 2016). In the interior, it increased to between 1.73‰ ± 0.15‰ and 2.95‰ ± 0.15‰. The rim sample shows a C/N ratio (7.5) that is slightly higher than the Redfield ratio at 6.63 (C:N = 106:16; Redfield, 1934), but is consistent with bulk cyanobacteria mats that produce significant amounts of EPS and sheaths (Berrendero *et al.*, 2016). Both interior samples show a C/N ratio (8–8.5), indicating a preferential loss of N-bearing organic molecules.

**Table 4.** Carbon and Nitrogen Isotope Data

Sample	[TOC] [%]	δ^13^C [‰]	SE (δ^13^C)	[N] [%]	δ^15^N [‰]	SE (δ^15^N)	C/N
ET17-L14_Rim_2a	0.38	−23.18	0.11	0.05	−1.72	0.15	7.5
ET17-L14_Interior_2a	0.86	−15.78	0.11	0.10	1.73	0.15	8.5
ET17-L14_Interior_2b	0.80	−13.77	0.11	0.10	2.95	0.15	8.0

SE for [TOC] is 0.17 and [N] 0.03, estimated based on repeated measurements of internal standards.

TOC, total organic carbon concentration; N, total organic nitrogen concentration; SE, standard error; C/N, carbon to nitrogen ratio of extracted organic matter.

### 3.7. High P-T alteration experiments on interior silicified cells

To investigate the degradation process beyond the current interior state of the palisade sinter sample at the Terrace Geyser, two types of thermal alteration experiments were performed: (1) short-duration, single-step alteration of untreated (wet) samples, and (2) long-duration, multi-step alteration of untreated (wet) and dried samples (for details see Section 2.7).

**FIG. 10.**
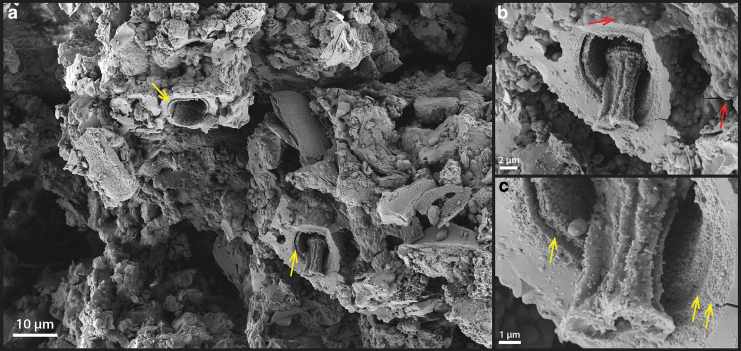
Single-step high T/P alteration experiment: 165°C, 72 h. **(a)** SEM overview image of the resulting palisade fabric after experiment. The general appearance of the interspace smaller filamentous structures is completely altered, relative to the unaltered interior sinter samples. Sheaths, as well as some structures resembling cellular material remained (arrows). **(b)** Zoom-in view of the sheath structure (from **a**) that contained a cell-like structure. Note the outer surface of sheath is covered by silica spheres up to 1 μm in diameter. **(c)** Zoom-in view of the cell-like material and sheath, showing that the sheath itself has fragmented into several concentric layers (arrows), whereas the inner-sheath material resembles preserved shrunken cells.

The short-duration, single-step experiments show clear evidence of early degradation. SEM images show that small filamentous structures coating the exterior of sheaths (as described in Section 3.3, see Fig. 7d, e, and g) are already lost at 165°C (Fig. 10a, b). The resulting filamentous sheaths show a general smooth surface texture outlined by enlarged silica spheres around 1 μm in size (Fig. 10b: arrows). The sheaths themselves display a double-layered or multi-layered morphology in the breakage plane (Fig. 10a,c: yellow arrows). This could possibly indicate a difference in resistance between two distinct textures: an inner, porous layer that initially contained silica spheres (Fig. 8c) and an outer, less porous layer that initially contained more flattened silica layers (Fig. 10c).

**FIG. 11.**
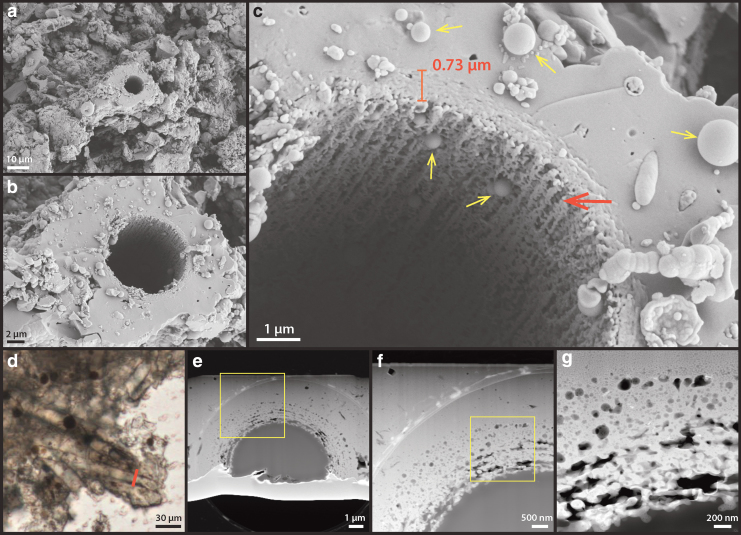
Single-step high T/P alteration experiment: 250°C, 72 h. **(a)** SEM overview image of the resulting palisade fabric after experiment. Remaining cell-like structures are not found at the interior of sheath. **(b)** Zoom-in view of the sheath structure (from **a**). **(c)** A further zoom-in image showing that the sheath structure has notably thinned, whereas significant porosity has developed from the interior of the sheath (red arrow). These pores preferentially shape along the filament direction. Accompanying the destruction of the sheath, up to micrometer-sized, perfectly spherical silica spheres are found around the structure (yellow arrow). **(d)** Optical microscope image from the experimentally altered sample showing the cut position of FIB-prepared TEM foils. **(e)** TEM overview image of the 100-nm thin foil, showing the morphology of the altered sheath structure. **(f)** Zoom-in image of the sheath. **(g)** Further zoom-in view of the sheath interior, showing the significantly degraded sheath and development of porosity within the structure.

Although most filaments appear hollow, some cellular structures were preserved within the sheaths. The cellular structures appear to have hollow interiors and collapsed, wrinkled exteriors (Fig. 10c). At 250°C, all cellular structures within the sheaths were lost (Fig. 11a–c) and the sheaths themselves appeared much thinner (<1 μm) and porous at their interior surfaces (Fig. 11c: red arrow). It appears that the less resistant, more porous interior layer of the sheaths has been destroyed, leaving only the less porous exterior layer. The pores in this exterior layer appear to align along the filament direction (Fig. 11c: red arrow). TEM analysis further confirmed that degradation occurred throughout the sheath structure (Fig. 11d–g), and that structural porosity has developed from within the sheath, resulting in its deterioration (Fig. 11g).

**FIG. 12.**
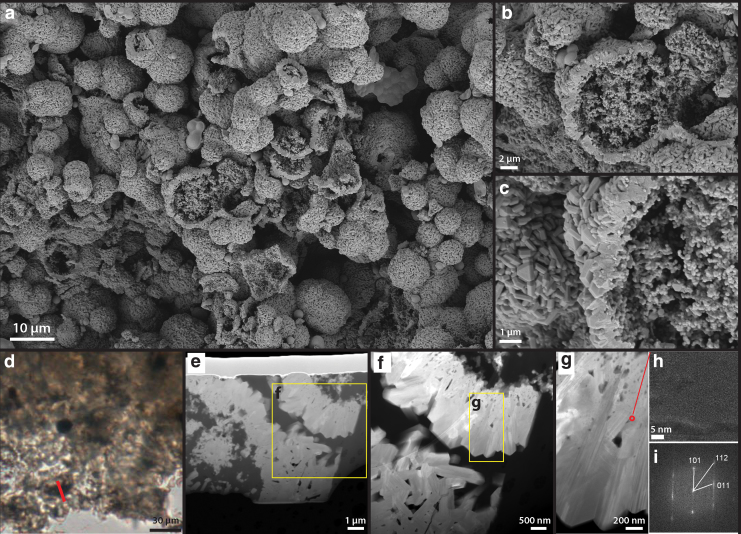
Single-step high T/P alteration experiment: 300°C, 72 h. **(a)** SEM overview image of the resulting material after experiment. Spherical ball-like structures have formed. **(b)** Zoom-in image of the spherical structure **(c)** A further zoom-in image of the spherical structure rim and interior, showing a crystalline rim and granular interior. **(d)** Optical microscope image from the experimentally altered sample showing the cut position of FIB-prepared TEM foils. **(e)** TEM overview image of the 100-nm thin foil, showing a cross-sectional view of the spherical structure. **(f)** Zoom-in image of the crystalline rim. **(g)** A further zoom-in view of the crystalline rim, showing blade-like structures. **(h)** High-resolution molecular structure of the crystal. **(i)** 2D Fourier Transform of the molecular structure map, from which the mineralogy of the crystal has been calculated. The mineral was determined to be α-cristobalite.

In comparison, the exterior, smooth silica casts were largely intact (Fig. 11c, i), preserving the external morphology of the sheath. SEM images also revealed perfect, up to micron-size silica spheres, occurring all around the sheath structures (Fig. 11c: yellow arrows). These types of silica spheres were not present at the lower temperature steps, indicating a different mechanism of formation possibly linked to silica dissolution and re-precipitation in pore fluids (Iler, 1979; Jones and Renaut, 2007: Figs. 10–13). At 300°C, the sinter texture self-organized into up to 10-μm-sized blocky lepispheres (Fig. 12a). These spherical structures have a well-defined, granular interior and a subangular crystalline rim (Fig. 12b, c). TEM analysis indicates that these rims have a mineralogy consistent with α-cristobalite (Fig. 12g–i).

**FIG. 13.**
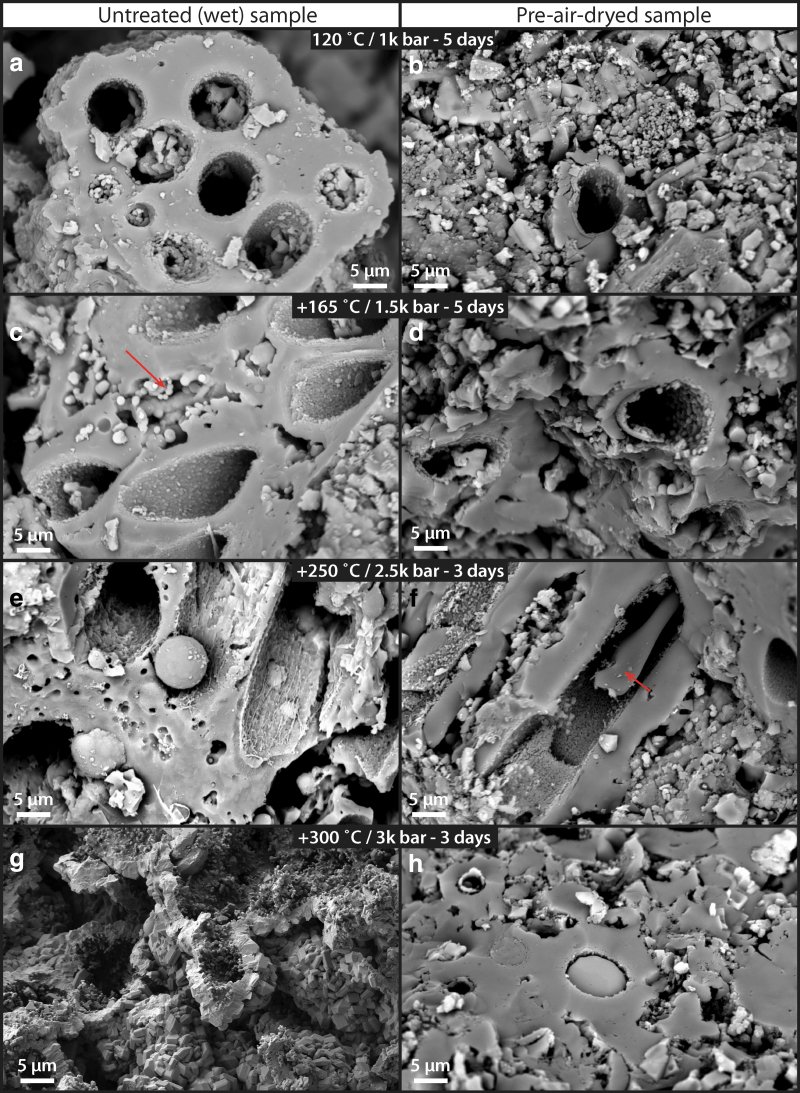
Multi-step high T/P alteration experiment (120–300°C, 1–2.5 kbar) of untreated (left column: **a, c, e,** and **g**) and pre-air-dried (right column: **b, d, f,** and **h**) samples. **(a, b)** 120°C, 1 kbar step. Both samples preserved primary sheath structure, although the air-dried sample experienced higher physical damage due to the effect of high pressure on a dryer sample. **(c, d)** 165°C, 1.5 kbar step. Both samples still preserved primary sheath structure. No significant degradation was observed at the sheath level from the previous step. **(e, f)** 250°C, 2.0 kbar step. The untreated sample showed significant damage of the sheath structure, whereas the predried sample showed no additional degradation from the previous heating step. Occasionally, inner sheath materials resembling degraded cellular organics have been preserved (arrow). **(g, h)** 300°C, 2.5 kbar step. The untreated sample showed formation of self-organized crystalline silica structures, whereas the predried sample did not transform and still preserved the original sheath structure.

Similar opal-CT lepispheres have been documented in long-term diagenesis experiments (Alleon *et al.*, 2016) as well as in natural hydrothermal fields (Rodgers *et al.*, 2002; Lynne *et al.*, 2005), relating this mineral formation to sinter–water interactions.

The second, multi-step progressive alteration experiments revealed more details in the degradation pathways. First of all, image analysis of an untreated (wet) set of samples (Supplementary Fig. S4) showed that at the 120°C step, 58% (*n* = 30) of sheath structures still contained some interior cellular structures, whereas this number reduced to 7% (*n* = 31) at the 165°C step, and 0% (*n* = 9) at the 250°C step. This trend demonstrates that the thermal destruction of morphologically identifiable cellular organics within the sheath structure is progressive in accordance to the temperature and pressure conditions of the experiments.

Even though the generally recognizable sheathed structures are still present at the 250°C step, most structural organics that would allow the identification of original cellular structures have been broken down. This result clearly demonstrates that the general morphology of silica-templated sheaths (and the overall palisade fabric) can survive diagenesis. Moreover, the direct comparisons between untreated (wet) and air-dried sets of samples show that the dry samples consistently preserve better at higher T/P conditions (Fig. 13). At the 250°C step, degradation was less pronounced in the dry samples and some cellular material appeared to be still preserved within the sheath (Fig. 13f: arrow). At the 300°C step, the primary silicified sheath fabric was again completely obliterated in the untreated (wet) sample (Fig. 13g), whereas the sheathed cellular fabric in the dry sample was still recognizable (Fig. 13h). These observations indicate that the amount of water within the sinter sample plays a significant role at degrading cellular organics and transforming the original silica-templated sheath fabric toward higher P/T conditions.

## 4. Discussion

This study employed a twofold approach toward investigating the formation of palisade structures occurring on the Terrace Geyser in the Upper Basin of El Tatio geothermal field: (i) a macro- and micro-scale structural, chemical, and molecular characterization of the palisade sinter rim, interior, and closely associated, living microbial mats; and (ii) experimental diagenesis of palisade material to investigate the preservation of cell structures under diagenetic conditions that cannot be observed in the field.

Although palisades are well known in geothermal settings, and previously described in other modern and ancient systems (Walter *et al.*, 1996; Lynne and Campbell, 2004; Campbell *et al.*, 2015), the primary goal of this work is to provide the first systematic description of palisade textures at the El Tatio geothermal field, which is a particularly dry and UV-rich environment. These highly unusual and extreme conditions make El Tatio an exceptionally relevant modern analogue for Precambrian evaporative environments.

### 4.1. Characterization of the sinter surface and closely associated microbial mats

The wetted parts of the outer rim of the terrace sinter appear to be colonized and dominated by sheathed, filamentous microorganisms (Figs. 1–3). The cells appear to be unbranching, are isodiametric, and lack features such as tapered trichomes, heterocysts, or akinetes; single trichomes occur within sheaths (Fig. 2d, e). These morphological features indicate the presence of the cyanobacterium *Leptolyngby* sp. (Castenholz *et al.*, 2001; Komárek, 2007). This species particularly thrives in extremely dry, evaporative settings (Wilmotte and Herdman, 2015).

This differs from many other hot spring settings worldwide where, at water temperatures below 35°C, the cyanobacterium *Calothrix* sp. is typically observed to be responsible for palisade fabric formation in sinter deposits (Konhauser *et al.*, 2003; Campbell *et al.*, 2015; Wilmotte and Herdman, 2015; Smythe *et al.*, 2016). Notably, although the color of the sheaths in Fig. 3b and c appears brown, which is a known characteristic of *Calothrix* sp., this color is not unique to, or diagnostic of *Calothrix* sp. The pigmentation seen here (Fig. 3b, c) indicates the presence of the pigment scytonemin, a common adaptation by numerous cyanobacterial taxa (incl. both *Leptolyngbya* sp. and *Calothrix* sp.) to intense UV radiation at the surface (Garcia-Pichel and Castenholz, 1991). The growth temperatures of *Leptolyngbya* sp. and *Calothrix* sp. overlap, although *Leptolyngbya* sp. is known to occur in much wider temperature regimes and diverse environments such as in desert, marine, and freshwater settings (Wilmotte and Herdman, 2015). These physiological adaptations are important at El Tatio, since extreme day**–**night temperature fluctuations (more than 30°C difference) occur and hot springs effluents are characterized by moderately saline Na-Cl type water (Cortecci *et al.*, 2005; Landrum *et al.*, 2009), as well as high evaporative cooling gradients (Nicolau *et al.*, 2014).

At the surface of the Terrace Geyser, the living cells of *Leptolyngbya* sp. (Figs. 1 and 3a), which still contain pigments such as carotenoids (β-carotene) and phycobilins (Fig. 3b, c), appear to be subjected to the first stage of silicification, as evidenced by small silica spheres that adhere to the outer EPS filamentous network of individual sheaths (Fig. 5). This provides strong evidence for the cyanobacterial sheaths to act as a template for early silicification, as has been proposed earlier (Konhauser *et al.*, 2004; Benning *et al.*, 2005; Smythe *et al.*, 2016). The whitish outer sinter rim, representing the areas where the terrace has evaporated to dryness, consists of a thin halite-rich silica crust (Supplementary Fig. S3) that is devoid of any cyanobacteria.

Immediately below the thin white outer layer of silica, sheathed cyanobacteria are observed (Fig. 6), likely representing the last generation of cyanobacteria growing at the surface before evaporation to dryness occurred. These sheathed cyanobacteria, however, have been degraded considerably, lacking internal cellular structures (Fig. 6d–f). Only the external sheath and occasionally some more resistant, cellular membrane structures are observed to be preserved (Fig. 6f). These membrane structures still possess a fluorescence signal for β-carotene, a common accessory pigment produced by cyanobacteria (Green and Parson, 2003; Beale, 2008).

Reproductive hormogonia were also identified in close proximity to the sheathed cyanobacteria (Fig. 6c: arrows), which are known to occur in *Leptolyngbya* sp. (Komárek, 2007). This may further indicate that the microorganisms were under considerable stress as the environment transitioned to a dry and evaporative condition, prompting them to reproduce rapidly to ensure continued colonization of the sinter surface when conditions once again would become wetter and more favorable for growth.

### 4.2. Sinter interior and the orientation of the palisade fabric

Toward the interior of the sinter deposit, the overall texture is characterized by lamina of dense green layers (1–5-mm thick) alternating with more porous, whitish color, and lower biomass layers (Fig. 2a, b). The microbial filament orientation within the dense green layer is vertical or subvertical, generally aligning with the local downflow direction (Fig. 2c–f).

In contrast, within the whitish layers, filaments are oriented horizontally and are outward facing (Fig. 2f). This observation suggests that the green layers, presumably formed during biologically productive phases of the sinter growth, are likely influenced by the physical forcing of the local fluid flow: The downward filament alignment with the flow may indicate that the filaments were not yet rigid, similar to “streamer” type fabrics that are commonly associated with higher energy channel flow environments (Fig. 3a; Jones *et al.*, 1998). On the other hand, the whitish layers, though containing less visible microbial components, still possess microbial filaments templating the sinter growth. The result is a type of porous sinter fabric containing more precipitated silica/salts, with the overall, outward growth direction matching that of the sinter.

We note here that the laminated, outward-growing palisade sinter described here at low-temperature terracettes of the Terrace Geyser, El Tatio may be unique among other palisade fabrics recorded in hot spring deposits around the world. In many hot spring systems such as in Yellowstone National Park, the United States, Krisuvik, Iceland and Taupo Volcanic Zone (TVZ), and New Zealand, upright positioning of filaments of the palisade fabrics (also termed “stratiform stromatolite”) has been associated with summer periods where biological growth typically outpaces the rate of silica precipitation (Walter, 1972, 1976; Jones *et al.*, 1998; Konhauser *et al.*, 2004). In these environments, upright palisade fabrics have been interpreted to form in association with optimal growth conditions, as vertical microbial pillar structures have been found in quiet ponds formed by sinter terracettes (Campbell *et al.*, 2015). Thus, the upright filament positioning is an intrinsic biological feature produced by the community of microorganisms. As also suggested by Campbell *et al.* (2015), the depositional stacking is likely controlled by a “pulse-pause” style of sheet flow that is sensitive to hot spring output conditions and lateral flow-path shifts (Campbell *et al.*, 2015). During the “pause” conditions, relative rates of silicification may increase, producing the type of solid sinter horizons that bound palisade fabrics from below and above (Campbell *et al.*, 2015). Another scenario to produce such a solid sinter horizon could be due to passive silica precipitation templating entirely on biologically produced EPS, if sufficient biomass is available (Handley *et al.*, 2005, 2008).

At the Terrace Geyser, however, these models of palisade fabric formation do not necessarily apply, because living cyanobacterial mats at low temperature (<40°C) conditions are rarely found growing in flat/quiet ponds. These communities only appear to colonize the rims of terracettes (Fig. 1). Here, these cyanobacterial mats have grown attached to vertical walls while experiencing faster fluid flows due to the vertical drop-off of the flow at the rim.

Regardless of detailed flow conditions, the high UV radiation of the Atacama Desert as well as the generally dry, salty, nutrient-poor, low/shifting temperature, and evaporative conditions may have combined to produce the challenging conditions that prevent microbial colonization from occurring on flat, horizontal surfaces.

### 4.3. Palisade fabric textural development

Biologically mediated production of extracellular sheaths of cyanobacteria can be viewed as a strategy to survive in high-rate silicification environments, even though the silicification process itself may be summarized as a predominately passive, abiotic process (Konhauser *et al.*, 2004; Benning *et al.*, 2005). Phoenix *et al.* (2002) has demonstrated that for their model organism, the cyanobacterium *Calothrix* sp. KC97, the extracellular sheath is mostly made of low-reactivity neutral sugars that also contain smaller amounts of carboxyl and amino groups (Phoenix *et al.*, 2002).

In a controlled experimental study where *Calothrix* sp. was grown in silica-supersaturated medium (300 ppm Si), the sheath structure was found to double or triple in thickness (Phoenix *et al.*, 2000). This observation indicated a clear connection between cyanobacterial extracellular sheath structure and dissolved silica in solution.

In our observations, silica spheres that are 50–100 nm in diameter silica spheres are found throughout the sheath structure (Figs. 5e and 8), indicating that the sheath itself is a composite material that contains both extracellular organic molecules and abiologically precipitated silica, which gives rise to its rigid and brittle material property (Figs. 3c and 5).

The internal structure of the sheath also appears to control the direction of silicification, allowing silica to only accumulate at the exterior of the sheath (Fig. 8a, b), as has already been indicated by Phoenix *et al.* (2002). This explains its protecting role against excess silicification, preventing moisture loss during dehydration, as well as shielding against high levels of UV radiation (Garcia-Pichel and Castenholz, 1991; Phoenix *et al.*, 2002, 2006; Rastogi and Incharoensakdi, 2014).

Since the sheaths of cyanobacteria are already semi-rigid due to early silicification (silica templating on external sheath; Fig. 5), it is unlikely that the sheaths themselves are sufficiently mobile to change orientation and position in response to day**–**night cycles. Therefore, we suggest that the relative orientations of filaments in our palisade sinter samples (laying parallel vs. perpendicular to lamination) do not record a behavioral adaptation in response to light (the phototaxis hypothesis). Although many *Leptolyngbya-* and *Calothrix*-like, sheathed filamentous cyanobacteria are not known to be mobile, their reproductive shorter segments of hormogonia cells generally possess gliding motility (Damerval *et al.*, 1991; Falkow *et al.*, 2006). These sheath-less hormogonia filaments were, indeed, identified in the outer rim as well as in the interior of the studied sinter samples (Fig. 6 and Supplementary Fig. S2; Fig. 7 and Supplementary Fig. S3), suggesting that the migration of hormogonia could represent a logical mechanism for the fabric development of the sinter.

The following model can, therefore, be envisioned: during the biologically productive phase of the sinter growth cycle (wet and favorable conditions); microbial filaments at the terrace rim are less rigid due to less precipitated silica present, and therefore are more likely to stay aligned to lamination with flow, draping downward. During dry conditions with high silica precipitation relative to biological growth, filaments grow outward (perpendicular to lamination), partially due to stressed cyanobacteria producing hormogonia cells trying to escape entombment, and partially due to precipitating silica providing a physical support for filaments to remain perpendicular to lamination. The particularly dry and evaporative conditions of the El Tatio region are, therefore, inducive for this type of changing water activity, explaining the common appearance of palisade fabrics in sinter deposits from active as well as extinct geysers in the area (Munoz-Saez *et al.*, 2016).

In addition to the change in filament orientation that characterizes the primary fabric of modern palisade sinters, palisade textures in the geological record are also characterized by their uniquely shaped pore structures (Campbell *et al.*, 2015). In our samples, as well as samples previously described at El Tatio from nearby extinct sinters (Munoz-Saez *et al.*, 2016), elongated pore structures are present and are, in general, aligned to the direction of microbial filaments (Fig. 2b, d, and f; Munoz-Saez *et al.*, 2016: Fig. 3). When microbial filaments are of high density and oriented flat to lamination, the porosity of the sinter is visibly low and uncharacteristic (Fig. 2). However, as soon as silica/mineral precipitation outpaces the rates of biological growth (low filament density), pore structures emerge and have a high tendency to align with pre-existing, perpendicularly aligned, silicified cyanobacterial sheaths (Fig. 2e, f).

These observations indicate that silicified, sheathed cyanobacteria can serve as a framework structure influencing the character of the sinter pore networks. These pore networks, when properly identified, may serve as a strong textural record that evidence the behavior and adaptation strategy of once-existing cyanobacteria. However, due to the high volume of porosity of the palisade fabric, during diagenesis, secondary infills or patchy diagenesis commonly occur and cause destruction of the original fabric, producing a type of “massive-mottled and diffusely layered fabric” (Campbell *et al.*, 2015). These unfavorable diagenetic conditions cause palisade fabrics—a rare find in deposits older than the Quaternary age, except in very old sinters where porosity is filled by very early hydrothermal silicification (Djokic *et al.*, 2017).

Therefore, in the search for biosignatures in hot spring deposits on the early Earth and Mars, silicification by early hydrothermal fluids may be a significant factor for preserving the detailed, morphologically diagnostic palisade textures. Priorities should be given to recognize and study such silicification processes.

### 4.4. Preservation of cell structures under diagenetic conditions

The silica-encapsulation and degradation of filamentous microbial mats observed in samples from the rim to the interior of the sinter terraces captures the important first steps in the formation of palisade texture in this particularly arid, evaporative environment. However, to investigate whether these palisade textures can be preserved during burial and early diagenesis, we artificially accelerated the degradation process through controlled experiments at elevated temperature and pressures.

Conditions for the first set of experiments were set at the same pressure (250 bar) but at varying temperatures, which were aimed at understanding how different components of the filamentous cyanobacteria (sheaths, trichomes) decay. The second set of experiments, which were designed to follow a realistic burial scenario after the modern geothermal gradient of the Earth (see Section 2.7), aimed at following the samples under progressively higher temperature and pressure conditions and more specifically to test the degradation mechanisms under wet and dry conditions.

The first set of experiments showed that when silica sinter (untreated, wet material) is subjected to elevated temperature and pressure—as would be the case during diagenesis associated with burial—the entrapped cyanobacteria are progressively degraded after a specific progression of steps. At 165°C (Fig. 10a), nonsheathed microorganisms are completely destroyed, whereas the sheaths themselves are preserved. Within the sheaths, occasionally cellular material can be preserved (Fig. 10b, c). The sheath structure appears to degrade into concentric shells (Fig. 10c, arrows), which is likely linked to shifts from internal spherical silica nanospheres to lenticular silica nanoparticles during growth (Fig. 8c). The inner sheath material has shrunk relative to the outer sheath layers during degradation (Fig. 10c, arrows). At 250°C, all cellular materials appear to have been degraded, leaving only hollow sheath structures (Fig. 11).

The sheath itself only consists of a much thinner outer layer, whereas the inner layers have been completely destroyed (Fig. 11c). This outer layer displays a distinct nanometer scale porosity (Fig. 11c, g) that likely represents the negative cast after destruction of much of the original EPS components of the sheath. At 300°C (Fig. 12), all features associated with the presence of cyanobacteria, including the silica casts of the sheaths, are completely destroyed. The silica phases dissolved and re-precipitated as small nucleating crystals that reorganized themselves into 5–15-μm spherical structures (Fig. 12a–c) that were identified as cristobalite (Fig. 12e–h). This artificial alteration step can be compared with the diagenetic conversion to opal-CT, that is commonly observed in older sinter deposits (Lynne and Campbell, 2004; Jones and Renaut, 2007; Campbell *et al.*, 2015). These results indicate that in closed environments, as is the case for these experiments when untreated samples are heated in sealed gold tubes, palisade fabric would not survive a metamorphic alteration step above 300°C.

The second set of experiments further showed that preservation is enhanced when the sinters are dried before diagenesis (Fig. 13), in line with previous artificial diagenesis experiments where a drying step was performed (Alleon *et al.*, 2017), suggesting that environmental conditions of silica precipitation as well as conditions directly after deposition are critical for the survival of a palisade fabric in the rock record. Water activity is known to greatly influence postdepositional silica diagenesis and play a significant role in transforming silica from an amorphous state to a crystalline state (Lynne and Campbell, 2004; Jones and Renaut, 2007). A major compositional difference between amorphous silica (opal-A), opal-C/T, and quartz is the amount of structurally bound water (Smith, 1998; Herdianita *et al.*, 2000). When water is heated under pressure toward the critical temperature (374°C), its physical and chemical behaviors change rapidly, which enable it to become more similar to an organic solvent (Clifford, 2007). Therefore, in diagenesis experiments such as ours, as well as in heated rocks under burial, dissolution caused by superheated water is likely a significant degradation pathway for the destruction and migration of organic matter. These effects may be clearly seen in our experiments where nanometer-scale porosity develops within the sheath structure that is likely caused by the degradation/dissolution of organic molecules (Fig. 11).

Consequently, we reason that depending on the specific conditions of diagenesis, particularly in open systems where dewatering can happen quickly enough during burial, preservation of organic molecules as well as morphological attributes of fossils should be greatly enhanced. Overall, similar to descriptions in other sinter deposits (Campbell *et al.*, 2001, 2015), these experiments have clearly shown that these cyanobacterial sheaths, being a composite organic-silica material, are most resistant to degradation and could be the only morphological evidence left that records the original cyanobacterial strategy to survive in silica sinter environments.

### 4.5. Biological implications on early degradation

Microbial community composition was investigated by using DNA amplicon sequencing and was used to describe the membership and relative abundances of microbial DNA present within the outer sinter rim, and the palisade sinter inside of the rim (interior), at the time of collection. The dominance of cyanobacteria observed during microscopic analysis of closely associated living microbial mats at the wetted sinter exterior suggested that abundant cyanobacteria DNA sequences would also be present in the sinter. However, a notably low abundance of cyanobacterial DNA (<0.4%), and a predominance of phylogenetically diverse sequences matching various heterotrophic bacteria (Fig. 9), demonstrates that early and rapid consumption and degradation of inner cellular features of the original cyanobacteria that formed the sheaths likely occurred after entombment in silica (Fig. 6).

Consistent with the morphologies observed in the living mats and residual sheath and preserved cells within the sinter (Figs. 3 and 6), the majority of cyanobacterial sequences were identified as *Leptolyngbya* sp. and *Plectonema* sp., both of which are sheath-forming members of the Type III Cyanobacteria (Table 3). In addition, *Synechocystis* sp., a unicellular cyanobacterium that does not form a sheath, were also present in the rim but not the interior, suggesting that they may be more susceptible to degradation than sheathed forms as sinter ages and new outer layers form.

Consistent with, and distinct from, the current findings, other types of sheath-forming cyanobacteria have been previously identified in such surface microbial mats in the past, such as *Lyngbya* sp., *Calothrix* sp., and *Phormidium* sp. (Phoenix *et al.*, 2006). These observations indicate that different species of cyanobacteria could be present in the closely associated habitats, together forming sheathed palisade fabrics. Regardless of taxonomic identity, all of these sheath-forming cyanobacteria-based communities are rapidly degraded toward the interior of the palisade sinter when entombed and buried under progressive sinter growth. The combined destruction of morphological features and biomolecules such as DNA prohibits a confident morphological identification of the original cyanobacterial species.

Our dataset suggests that, accompanying the degradation of original cyanobacterial community, an entirely different ecosystem of heterotrophic microorganisms takes hold during the degradation of the cellular material, including the nucleotides of DNA, which are often among the first cellular components degraded and reincorporated by living organisms in ecosystems. For example, *Algiphilus*, an obligate aerobic bacterium (Gutierrez *et al.*, 2012), is, in part, facilitating the degradation of original cyanobacteria in the rim. Then in the interior, anaerobic microorganisms are taking over, such as *Opitutus*, an anaerobic, polysaccharide-degrading organism (van Passel *et al.*, 2011).

Carbon and nitrogen stable isotopic analysis further supports this biological degradation mechanism of the original cyanobacteria within the palisade sinter. The increase in nitrogen isotope fractionation (δ^15^N) by 3–5‰ toward the sinter interior is typically observed when the primary organic matter has been degraded by heterotrophs that operate by aerobic respiration, increasing a trophic level (Minagawa and Wada, 1984; Adams and Sterner, 2000; Steffan *et al.*, 2015), confirming the presence of heterotrophic communities as shown from our DNA community analysis (Fig. 9). On the other hand, the increase in δ^13^C by 8–10‰ from the rim to the interior (8–10 mm apart; Fig. 2a) can be explained by selective preservation of specific organic carbon pools (Macko and Estep, 1984; Fry and Sherr, 1989).

Cyanobacterial sugar biosynthesis (leading to the production of EPS and sheaths) has been found to incorporate less of the lighter carbon ^12^C in comparison to the biosynthesis of lipids and other inner cellular organics (van Dongen *et al.*, 2002; van der Meer *et al.*, 2003). Our observations showed that the inner-sheath cellular organics of the original cyanobacteria have been rapidly degraded by aerobic heterotrophy (Figs. 6 and 7), which supports the idea that these isotopically lighter carbon components are preferentially being consumed and respired. Heavier organic carbon contained within the sheath structure appears to be protected by silicification, suggesting that the preservation of sheaths should further increase δ^13^C, leading to the 8–10‰ rise observed in our samples. Overall, these trends of rapid changes in carbon and nitrogen isotope signals record an ecological shift in the biological communities and could be used as a diagnostic tool in studying palisade sinter deposits.

## 5. Conclusions

In summary, using combined field sampling, DNA sequencing, isotopic analysis, and experimental approaches, this study provides a detailed account on the formation and progressive degradation mechanisms of palisade fabrics from the active hot spring system of El Tatio, Chile. The particularly evaporative environment of the sinter and its varying wet**–**dry cycles offered a unique opportunity to investigate the formation of these sinter fabrics in accordance with changing environmental conditions.

We conclude that the mechanism for palisade fabric development is intimately linked to the wet**–**dry cycles of the sinter, and that the initial degradation of the sheathed cyanobacteria is biological and rapid. After burial, we experimentally demonstrated that the sheath structure, owing to its incorporation of amorphous silica, is more resistant to diagenesis than the original cellular organics. The sheathed fabrics, as well as their associated primary porosity could be the most resistant feature left in the rock record. Consequently, this study provides a unique perspective on interpreting biosignatures left in ancient environments on our own planet as well as potentially for future astrobiological surveys on Mars and other worlds.

## Supplementary Material

Supplemental data

Supplemental data

Supplemental data

Supplemental data

Supplemental data

Supplemental data

## Abbreviations Used

CLSM: confocal laser scanning microscopy
DIC: differential interference contrast
EDX: energy dispersive x-ray spectroscopy
EPS: extracellular polymeric substances
FIB: focused ion beam
HAADF: high-angle annular dark-field
SEM: scanning electron microscopy
TEM: transmission electron microscopy
UV: ultraviolet
