# Supplementary material for: Formation and Preservation of Microbial Palisade Fabric in Silica Deposits from El Tatio, Chile
Source: Astrobiology. 2020 Mar 25;20(4):500–24. doi: 10.1089/ast.2019.2025 (PMC7133459; doi:10.1089/ast.2019.2025)
Supplement: Supplemental data [file Supp_Data1.pdf]

Supplementary Data

Supplementary Data S1

1. Confocal Laser Scanning Microscopy Methods

The photosynthetic apparatus of cyanobacteria is a thylakoid-membrane-bound complex that utilizes various pigments such as chlorophyll, phycobilins, carotenoids, and various binding proteins to achieve its intended energy conversion role (Grossman *et al.*, 1995; Green and Durnford, 1996). When pigments are bound to proteins as complexes, their optical absorbance as well as fluorescence properties change (Falkowski and Kiefer, 1985). Consequently, *in vivo* optical measurements on cyanobacterial cells cannot be taken as the sum of individual pigments, even though these pigments have been studied in detail through purification by solvent-extraction. Environmental factors as well as cell physiology have also been shown to alter the optical properties of the photosynthetic apparatus (Schubert and Hagemann, 1990; Campbell *et al.*, 1998). Therefore, the identification of the auto-florescence source from cyanobacteria can be a complex problem, frequently requiring making measurements at liquid nitrogen temperatures (at this temperature, the pigment–pigment, pigment–protein interactions are minimized) (Kühlbrandt *et al.*, 1994; Beale, 2008; Lamb *et al.*, 2015). Recent technological advances in confocal laser scanning microscopy, however, have offered new insights regarding the *in vivo* fluorescence signal from cyanobacteria (Roldán *et al.*, 2004; Vermaas *et al.*, 2008). It has been shown that a 488-nm laser primarily excites the phycobilins and carotenoids in *Synechocystics* sp. PCC 6803 rather than chlorophyll-a, giving unique insights on how these pigments are spatially distributed in cyanobacterial cells *in vivo* (Vermaas *et al.*, 2008). Consequently, the 488-nm laser line was used extensively in this study to characterize cells from the free-living state (at the exterior of the sinter environment) until the fossilized state (at the interior of the sinter up to about 10-mm depth).

At room temperature, the chlorophyll and phycobilisome pigments in cyanobacteria have a fluorescent emission peak between 640- and 700 nm (Vermaas *et al.*, 2008). Known pigments in this class and their emission maximums are phycocyanin (650 nm), allophycocyanin (665 nm), and allophycocyanin-B (675 nm) (Bittersmann and Vermaas, 1990; Sobiechowska-Sasim *et al.*, 2014). On the other hand, carotenoids are accessory pigments that help channel light energy into primary pigments such as chlorophyll, expanding the overall photoreception range (Green and Parson, 2003). A second role for carotenoids is photooxidation protection against excess visible and ultraviolet light radiation (Wada *et al.*, 2013). Known carotenoids and their emission maximums are β-carotene (560 nm), rhodopin (560–600 nm), and spheroidenone (560–620 nm) (Gillbro and Cogdell, 1989). These emission characteristics are used in this study for the interpretation of organic pigments present in our samples.

Supplementary References

Beale, S.I. (2008) Photosynthetic pigments: perplexing persistent prevalence of 'superfluous' pigment production. *Curr Biol* 18:R342–R343.

Bittersmann, E. and Vermaas, W. (1990) Fluorescence Lifetime Studies of Cyanobacterial Photosystem II Mutants. In *Current Research in Photosynthesis: Proceedings of the VIIIth International Conference on Photosynthesis Stockholm, Sweden, August 6–11, 1989,* edited by M. Baltscheffsky. Springer Netherlands, Dordrecht, pp 667–670.

Campbell, D., Hurry, V., Clarke, A.K., Gustafsson, P., and Öquist, G. (1998) Chlorophyll fluorescence analysis of cyanobacterial photosynthesis and acclimation. *Microbiol Mol Biol Rev* 62: 667–683.

Falkowski, P. and Kiefer, D.A. (1985) Chlorophyll a fluorescence in phytoplankton: relationship to photosynthesis and biomass. *J Plankton Res* 7:715–731.

Gillbro, T. and Cogdell, R.J. (1989) Carotenoid fluorescence. *Chem Phys Lett* 158:312–316.

Green, B. and Parson, W.W. (eds.) (2003) *Light-Harvesting Antennas in Photosynthesis*. Advances in Photosynthesis and Respiration. Springer Science+Business Media, Dordrecht, The Netherlands.

Green, B.R. and Durnford, D.G. (1996) The chlorophyll-carotenoid proteins of oxygenic photosynthesis. *Annu Rev Plant Physiol Plant Mol Biol* 47:685–714.

Grossman, A.R., Bhaya, D., Apt, K.E., and Kehoe, D.M. (1995) Light-harvesting complexes in oxygenic photosynthesis: diversity, control, and evolution. *Annu Rev Genet* 29:231–288.

Kühlbrandt, W., Wang, D.N., and Fujiyoshi, Y. (1994) Atomic model of plant light-harvesting complex by electron crystallography. *Nature* 367:614–621.

Lamb, J., Forfang, K., and Hohmann-Marriott, M. (2015) A practical solution for 77 K fluorescence measurements based on LED excitation and CCD array detector. *PLoS One* 10:e0132258.

Roldán, M., Thomas, F., Castel, S., Quesada, A., and Hernández-Mariné, M. (2004) Noninvasive pigment identification in single cells from living phototrophic biofilms by confocal imaging spectrofluorometry. *Appl Environ Microbiol* 70:3745–3750.

Schubert, H. and Hagemann, M. (1990) Salt effects on 77k fluorescence and photosynthesis in the Cyanobacterium Synechocystis sp. PCC 6803. *FEMS Microbiol Lett* 71:169–172.

Sobiechowska-Sasim, M., Stoń-Egiert, J., and Kosakowska, A. (2014) Quantitative analysis of extracted phycobilin pigments in Cyanobacteria—an assessment of spectrophotometric and spectrofluorometric methods. *J Appl Phycol* 26:2065–2074.

Vermaas, W.F.J., Timlin, J.A., Jones, H.D.T., Sinclair, M.B., Nieman, L.T., Hamad, S.W., Melgaard, D.K., and Haaland, D.M. (2008) In vivo hyperspectral confocal fluorescence imaging to determine pigment localization and distribution in cyanobacterial cells. *Proc Natl Acad Sci U S A* 105:4050–4055.

Wada, N., Sakamoto, T., and Matsugo, S. (2013) Multiple roles of photosynthetic and sunscreen pigments in cyanobacteria focusing on the oxidative stress. *Metabolites* 3:463–483.
