# Supplementary material for: Formation and Preservation of Microbial Palisade Fabric in Silica Deposits from El Tatio, Chile
Source: Astrobiology. 2020 Mar 25;20(4):500–24. doi: 10.1089/ast.2019.2025 (PMC7133459; doi:10.1089/ast.2019.2025)
Supplement: Supplemental data [file Supp_Datas2-3.pdf]

Supplementary Data S2

1. Artificial Diagenesis Experiments

Conditions of experimental temperature and pressure conditions follow Supplementary Tables S2-1 and S2-2. Supplementary Table S2-1 reflects the short-term experiment that heat samples directly to target temperature and pressure conditions. Supplementary Table S2-2 reflects progressive heating experiments in which identical sets of samples were heated progressively to higher temperature and pressure conditions, while a subset of samples were retrieved at each step and analyzed.

Experiment #2 temperature and pressure calculations were made by assuming a constant geothermal gradient of 25°C temperature rise per kilometer depth. Starting room temperature was assumed at 30°C at the surface (1 bar). Then, the pressure increase per kilometer depth was calculated by assuming the whole-rock density of 3000 kg/m^3. This gives a slope of 294 bar pressure increase per kilometer depth (corresponding to the 25°C geothermal gradient).

Supplementary Table S2-1. Experiment #1 (Direct Heating) Autoclave Temperature and Pressure Conditions.

Temperature (°C)	*Pressure (bar)*	*Duration (h)*
165	250	72
250	250	72
300	250	72

Supplementary Table S2-2. Experiment #2 (Progressive Heating) Autoclave Temperature and Pressure Conditions at Each Step

Temperature (°C)	*Pressure (bar)*	*Duration (h)*
120	1059	120
165	1589	120
250	2588	72
300	3176	72

Supplementary Data S3

Phylum-level taxonomy summary of palisade samples ET17-L14_Rim and ET17-L14_Interior

	ET17-L14_Rim		*ET17-L14_Interior*	
Phylum-level taxonomy	*Count*	*%*	*Count*	*%*
Archaeal phyla	2	0.003	4	0.01
Acidobacteria	22	0.03	39	0.05
Actinobacteria	3	0.003	3	0.003
Bacteroidetes	18,483	24.5	23,527	30.8
Balneolaeota	59	0.08	71	0.09
Chlamydiae	11	0.01	7	0.01
Chlorobi	27	0.04	22	0.03
Chloroflexi	315	0.42	338	0.4
Cyanobacteria	294	0.39	241	0.3
Deferribacteres	5	0.01	4	0.01
Deinococcus-Thermus	25	0.03	38	0.05
Firmicutes	142	0.19	78	0.1
Fusobacteria	0	0	1	0.001
Gemmatimonadetes	183	0.2	116	0.2
Ignavibacteriae	19	0.03	26	0.03
Nitrospinae	21	0.03	20	0.03
Nitrospirae	21	0.03	69	0.09
Planctomycetes	6140	8.1	3893	5.09
Proteobacteria	43,807	58.0	32,344	42.3
Spirochaetes	21	0.03	28	0.04
Tenericutes	4	0.01	24	0.03
Verrucomicrobia	5892	7.8	15,618	20.4
Total	75,490	100	76,509	100
