# Supplementary material for: Formation and Preservation of Microbial Palisade Fabric in Silica Deposits from El Tatio, Chile
Source: Astrobiology. 2020 Mar 25;20(4):500–24. doi: 10.1089/ast.2019.2025 (PMC7133459; doi:10.1089/ast.2019.2025)
Supplement: Supplemental data [file Supp_Fig1.pdf]

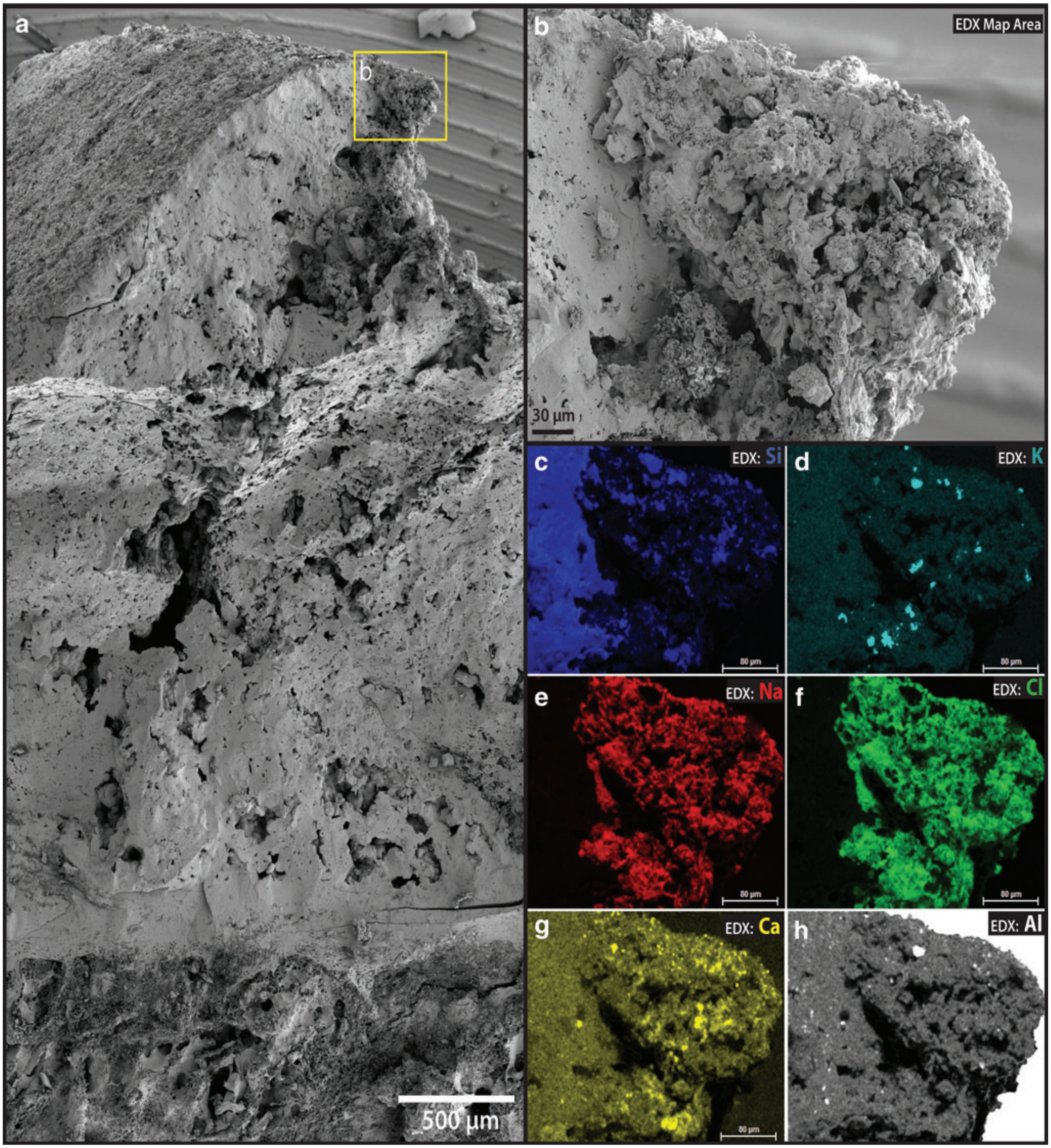

SUPPLEMENTARY FIG. S1. SEM image and EDX maps of the palisade sinter rim. **(a)** Overview SEM image. **(b)** Region for EDX analysis. **(c)** Si-map, **(d)** K-map, **(e)** Na-map, **(f)** Cl-map, **(g)** Ca-map, **(h)** Al-map. EDX, energy dispersive x-ray spectroscopy; SEM, scanning electron microscopy.
