# Supplementary material for: Formation and Preservation of Microbial Palisade Fabric in Silica Deposits from El Tatio, Chile
Source: Astrobiology. 2020 Mar 25;20(4):500–24. doi: 10.1089/ast.2019.2025 (PMC7133459; doi:10.1089/ast.2019.2025)
Supplement: Supplemental data [file Supp_Fig2.pdf]

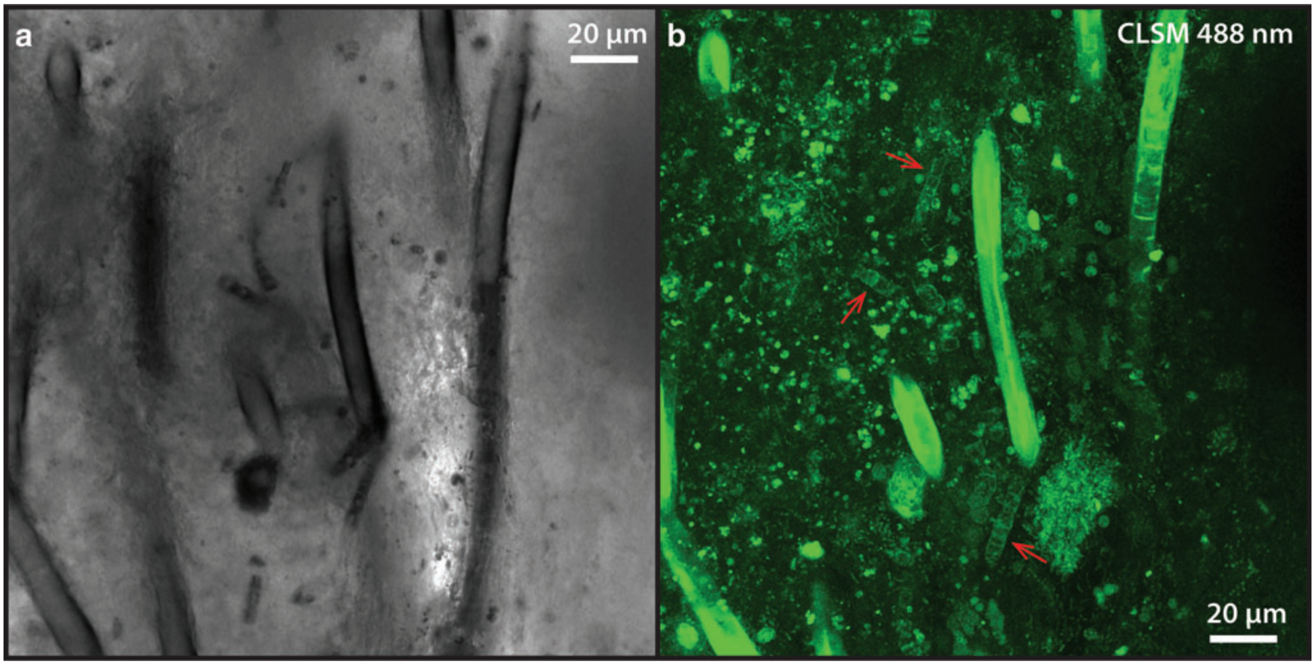

SUPPLEMENTARY FIG. S2. Optical DIC and CLSM image of microbial communities in the sinter rim. **(a)** Optical DIC image. **(b)** CLSM image showing that both the sheathed cyanobacteria and unsheathed shorter segments of filamentous cells fluorescence. In addition, other unicellular microbial communities containing similar fluorescent pigments might be present as well, shown as green dots in the image.
