# Supplementary material for: Formation and Preservation of Microbial Palisade Fabric in Silica Deposits from El Tatio, Chile
Source: Astrobiology. 2020 Mar 25;20(4):500–24. doi: 10.1089/ast.2019.2025 (PMC7133459; doi:10.1089/ast.2019.2025)
Supplement: Supplemental data [file Supp_Fig3.pdf]

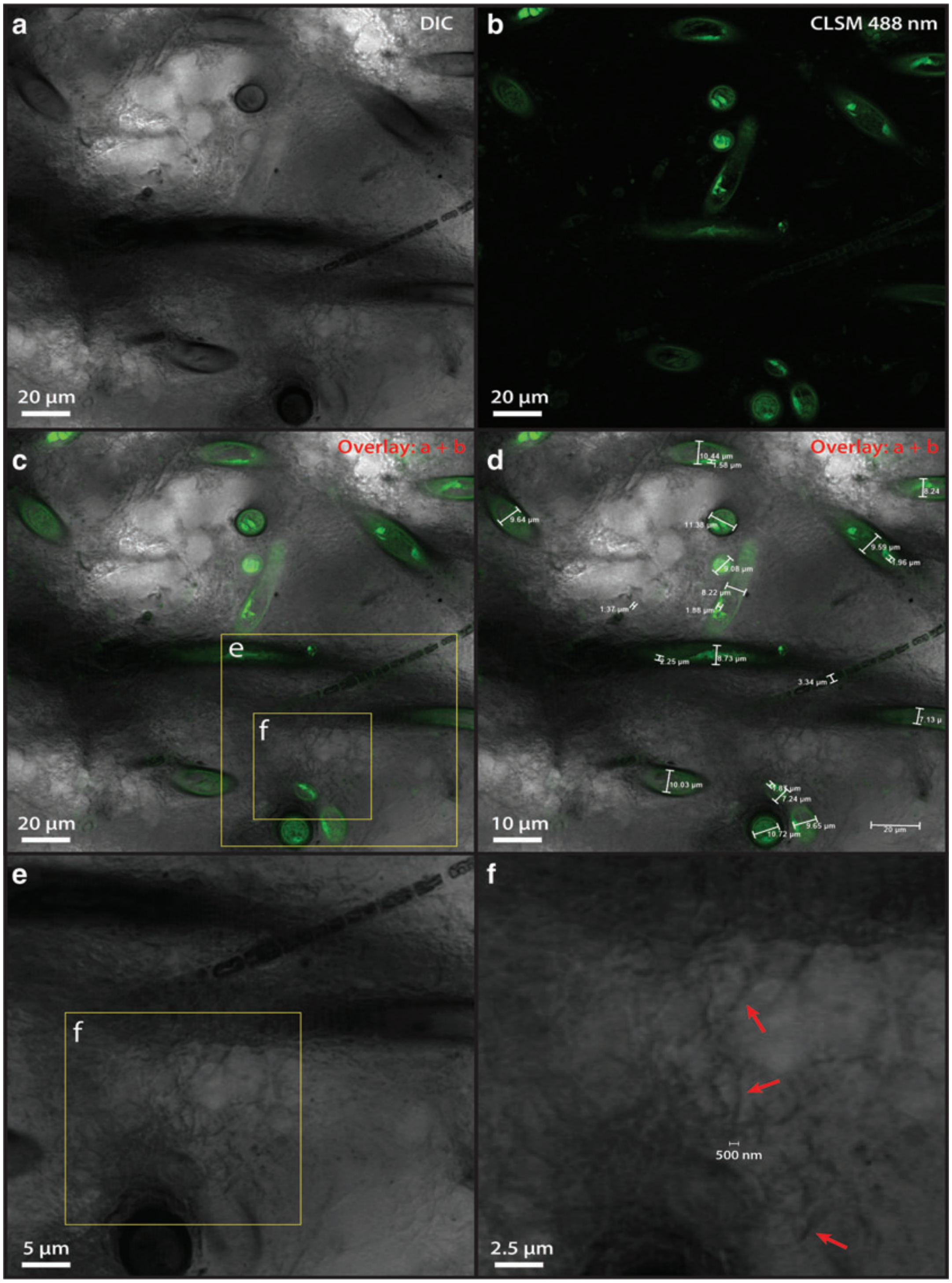

SUPPLEMENTARY FIG. S3. Optical DIC and CLSM image of microbial communities in the sinter interior. **(a)** Optical DIC image. **(b)** CLSM image showing that sheathed as well as unsheathed filamentous cells fluoresce. **(c)** Overlay of panel **(a, b,** and **d)** Overlay of panel **(a)** and **(b)**, with size measurements of sheath, internal shrunken filaments, as well as unsheathed cells displayed. **(e)** Zoom-in image from **(c, f)** A further zoom-in image from panel **(e)**, showing <500-nm diameter filamentous structures that do not fluoresce in response to the 488-nm laser (arrows). **(e, f)** 250°C, 2.0 kbar step. CLSM, confocal laser scanning microscopy; DIC, differential interference contrast.
