# Supplementary material for: Formation and Preservation of Microbial Palisade Fabric in Silica Deposits from El Tatio, Chile
Source: Astrobiology. 2020 Mar 25;20(4):500–24. doi: 10.1089/ast.2019.2025 (PMC7133459; doi:10.1089/ast.2019.2025)
Supplement: Supplemental data [file Supp_Fig4.pdf]

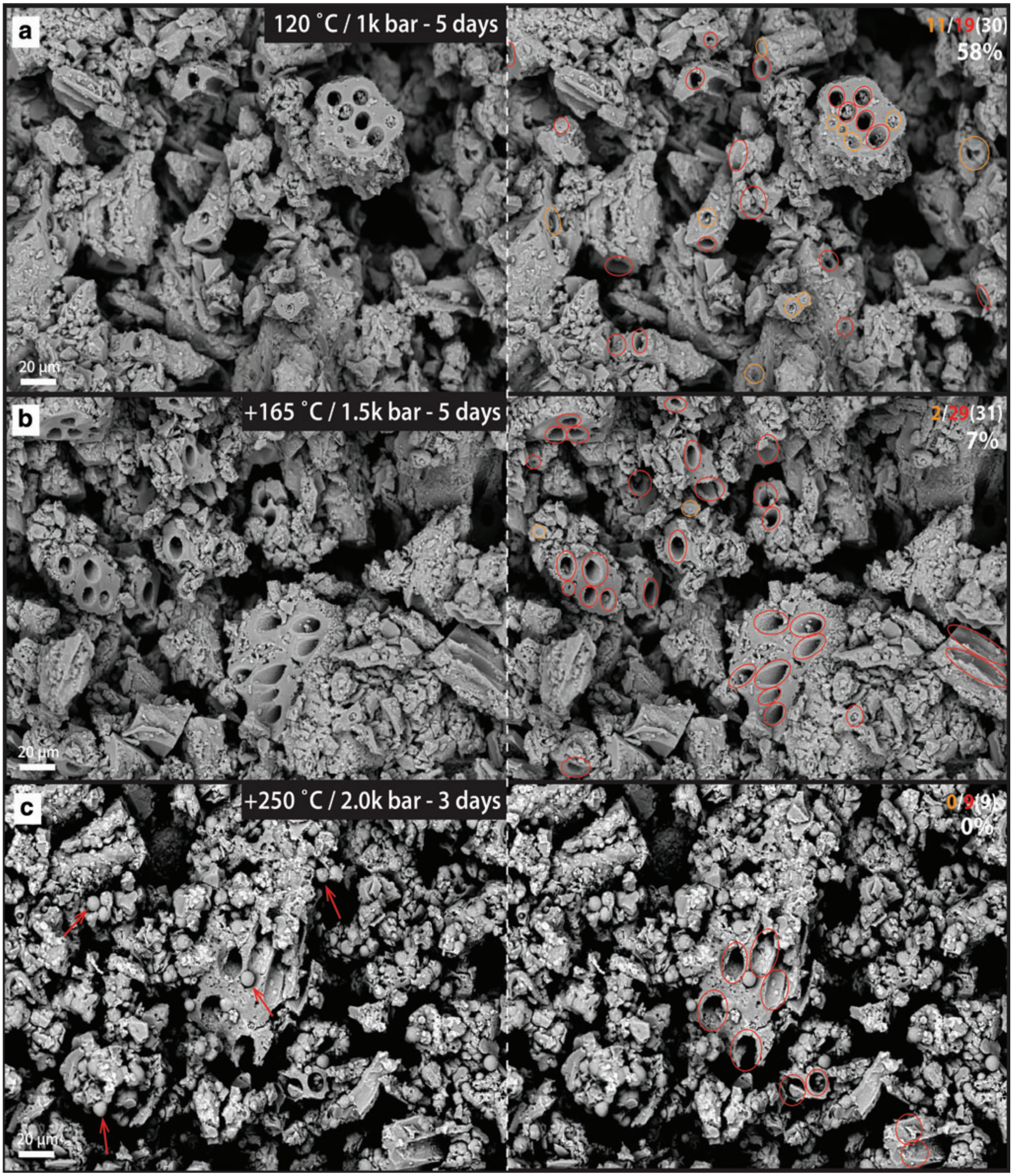

SUPPLEMENTARY FIG. S4. Multi-step high T/P alteration experiment (120–250°C, 1–2 kbar) of untreated samples with detailed analysis regarding preservation of cellular material within sheaths. **(a)** 120°C, 1 kbar step: 58% of sheaths that contained identifiable cellular material ($n = 30$). **(b)** 165°C, 1.5 kbar step: 7% of sheaths that contained identifiable cellular material ($n = 31$). **(c)** 250°C, 2.0 kbar step: 0% of sheaths that contained identifiable cellular material.
